# Genome and catabolic subproteomes of the marine, nutritionally versatile, sulfate-reducing bacterium *Desulfococcus multivorans* DSM 2059

**DOI:** 10.1186/s12864-016-3236-7

**Published:** 2016-11-15

**Authors:** Marvin Dörries, Lars Wöhlbrand, Michael Kube, Richard Reinhardt, Ralf Rabus

**Affiliations:** 1 General and Molecular Microbiology, Institute for Chemistry and Biology of the Marine Environment (ICBM), Carl von Ossietzky University Oldenburg, Oldenburg, Germany; 2 Institute of Forest Genetics, Johann Heinrich von Thünen Institute, Waldsieversdorf, Germany; 3 Max Planck Genome Centre Cologne, Köln, Germany; 4 Max Planck Institute for Marine Microbiology, Bremen, Germany

**Keywords:** Genome, Proteome, Metabolic reconstruction, Degradation pathways, Energy metabolism, Membrane proteins, Sulfate-reducing bacteria, *Desulfococcus multivorans*, *Desulfobacteraceae*

## Abstract

**Background:**

Sulfate-reducing bacteria (SRB) are key players of the carbon- and sulfur-cycles in the sediments of the world’s oceans. Habitat relevant SRBs are often members of the *Desulfosarcina*-*Desulfococcus* clade belonging to the deltaproteobacterial family of *Desulfobacteraceae*. Despite this environmental recognition, their molecular (genome-based) physiology and their potential to contribute to organic carbon mineralization as well as to adapt to changing environmental conditions have been scarcely investigated. A metabolically versatile representative of this family is *Desulfococcus multivorans* that is able to completely oxidize (to CO_2_) a variety of organic acids, including fatty acids up to C_14,_ as well as aromatic compounds.

**Results:**

In this study the complete 4.46 Mbp and manually annotated genome of metabolically versatile *Desulfococcus multivorans* DSM 2059 is presented with particular emphasis on a proteomics-driven metabolic reconstruction. Proteomic profiling covered 17 substrate adaptation conditions (6 aromatic and 11 aliphatic compounds) and comprised 2D DIGE, shotgun proteomics and analysis of the membrane protein-enriched fractions. This comprehensive proteogenomic dataset allowed for reconstructing a metabolic network of degradation pathways and energy metabolism that consists of 170 proteins (154 detected; ~91 % coverage). Peripheral degradation routes feed via central benzoyl-CoA, (modified) β-oxidation or methylmalonyl-CoA pathways into the Wood-Ljungdahl pathway for complete oxidation of acetyl-CoA to CO_2_. Dissimilatory sulfate reduction is fueled by a complex electron transfer network composed of cytoplasmic components (e.g., electron transfer flavoproteins) and diverse membrane redox complexes (Dsr, Qmo, Hmc, Tmc, Qrc, Nuo and Rnf). Overall, a high degree of substrate-specific formation of catabolic enzymes was observed, while most complexes involved in electron transfer appeared to be constitutively formed.

**Conclusions:**

A highly dynamic genome structure in combination with substrate-specifically formed catabolic subproteomes and a constitutive subproteome for energy metabolism and electron transfer appears to be a common trait of *Desulfobacteraceae* members.

**Electronic supplementary material:**

The online version of this article (doi:10.1186/s12864-016-3236-7) contains supplementary material, which is available to authorized users.

## Background

The decomposition of organic matter by sulfate-reducing bacteria (SRB) in anoxic (O_2_-depleted) marine sediments plays a major role in the global cycles of carbon and sulfur. This process accounts for more than 50 % of the total organic carbon (C_org_) mineralization in marine shelf sediments [1, 2] which are characterized by shallow waters and highest C_org_ input. The observed high process rates were early on proposed to demand complete substrate oxidation to CO_2_ [3]. The intensively studied deltaproteobacterial *Desulfovibrio* spp., however, oxidize organic substrates only incompletely to acetyl-CoA and possess only a rather limited substrate range and may, therefore, not be responsible for these rates. In contrast, members of the likewise deltaproteobacterial family *Desulfobacteraceae* are capable of complete oxidation and are nutritionally versatile [4]. Their substrate spectra range from readily degradable simple fermentation endproducts via long-chain fatty acids to more challenging molecules such as aromatic compounds and hydrocarbons [4]. Biogeographic investigations of various marine sediments revealed members of the *Desulfosarcina-Desulfococcus* clade (DSS) within *Desulfobacteraceae* to dominate the SRB community [5, 6]. Members of the family *Desulfobacteraceae* have long been known to dominate bacterial populations in marine shelf sediments (e.g., [7–10]) and were recently also detected in a sedimental sulfate methane transition zone [11] as well as an oxygen minimum zone off the coast of Namibia [12]. Next to their ecophysiological relevance for the biogeochemistry of marine environments, interest in SRB also arises from their long evolutionary history and their energy metabolism operating at the thermodynamic limit [13]. The first members of the *Desulfobacteraceae* to have their genomes sequenced are facultatively chemolithoautotrophic *Desulfobacterium autotrophicum* HRM2 [14], aromatic compound degradation specialist *Desulfobacula toluolica* Tol2 [15] and the two *n*-alkane degraders *Desulfococcus oleovorans* Hxd3 (unpublished) and *Desulfatibacillum alkenivorans* AK-01 [16]. Studies on the differential proteomic level have been performed with *D. autotrophicum* HRM2 [17, 18] and *D. toluolica* Tol2 [15].

The present study extends our current knowledge on *Desulfobacteraceae* by reporting the first complete genome of a *Desulfosarcina-Desulfococcus* clade member, the nutritionally versatile *Desulfococcus multivorans* (Table 1). Moreover, we advance the genome-based metabolic reconstruction of *D. multivorans* by differential proteomic analysis of cells adapted to 17 different substrate conditions.

**Table 1:** Properties of genome-sequenced representatives of completely oxidizing SRB

	Members of *Desulfobacteraceae*					
Property	*Desulfococcus multivorans* 1be1	*Desulfococcus biacutus* KMRActS	*Desulfococcus oleovorans* Hxd3	*Desulfatibacillum alkenivorans* AK-01	*Desulfobacula toluolica* Tol2	*Desulfobacterium autotrophicum* HRM2
Physiological characteristics						
Organic substrates						
Aliphatic hydrocarbons	-	-	C_12_ - C_20_	C_13_ - C_18_	-	-
Aromatic hydrocarbons	-	-	-	-	1	-
Fatty acids	< C_14_^a, b^	< C_7_^a^	C_4_ - C_18_	< C_16_	< C_4_	< C_16_^a^
Other aliphatic compounds	6	8	-	3 ^c^	8	8
Polar aromatic compounds	7	-	-	-	7	-
Electron acceptors						
Sulfate	+	+	+	+	+	+
Sulfite	+	+	+	+	+	-
Thiosulfate	+	+	+	+	+	+
Autotrophy	-	-	-	+	-	+
Syntrophy	-	n.d.	n.d.	+	n.d.	n.d.
Genome features						
Size (bp)	4 455 399	5 242 029	3 944 167	6 517 073	5 197 905	5 589 073
Sequencing status	complete	complete^d^	complete	complete	complete	complete
G + C content (mol%)	57	58	57	56	42	49
rRNA operons	3	1	1	2	4	6
tRNAs	54	51	47	56	48	50
Coding sequences (CDS)	3942	4708	3267	5296	4375	4943
Coding (%)	87	89	88	87	87	88
Average size (bp)	985	987	1066	1080	1039	1017
Assigned function	2648	3520	2602	4053	3188	3477
Conserved unknown	689	1162^e^	665^e^	1243^e^	681	1466^e^
Unknown	605				512	
Plasmids	-	-	-	-	-	1
Phage	-	n.d.	-	-	1	1
CRISPR	6	8	2	1	3	1
Accession number	CP015381	125602^g^	CP000859	CP001322	FO203503	CP001087
References	([31]; this study)	([75, 76]; IMG^f^)	([77]; IMG^f^)	[16, 78]	[15, 79]	[14, 80]

n.d., no data available

^a^also branched-chain fatty acids

^b^also cyclohexane carboxylate

^c^poorly utilized

^d^draft sequence

^e^without predicted function (https://img.jgi.doe.gov/)

^f^ IMG, https://img.jgi.doe.gov/

^g^NCBI taxon ID

## Results and discussion

### General genome features

**Fig. 1:**
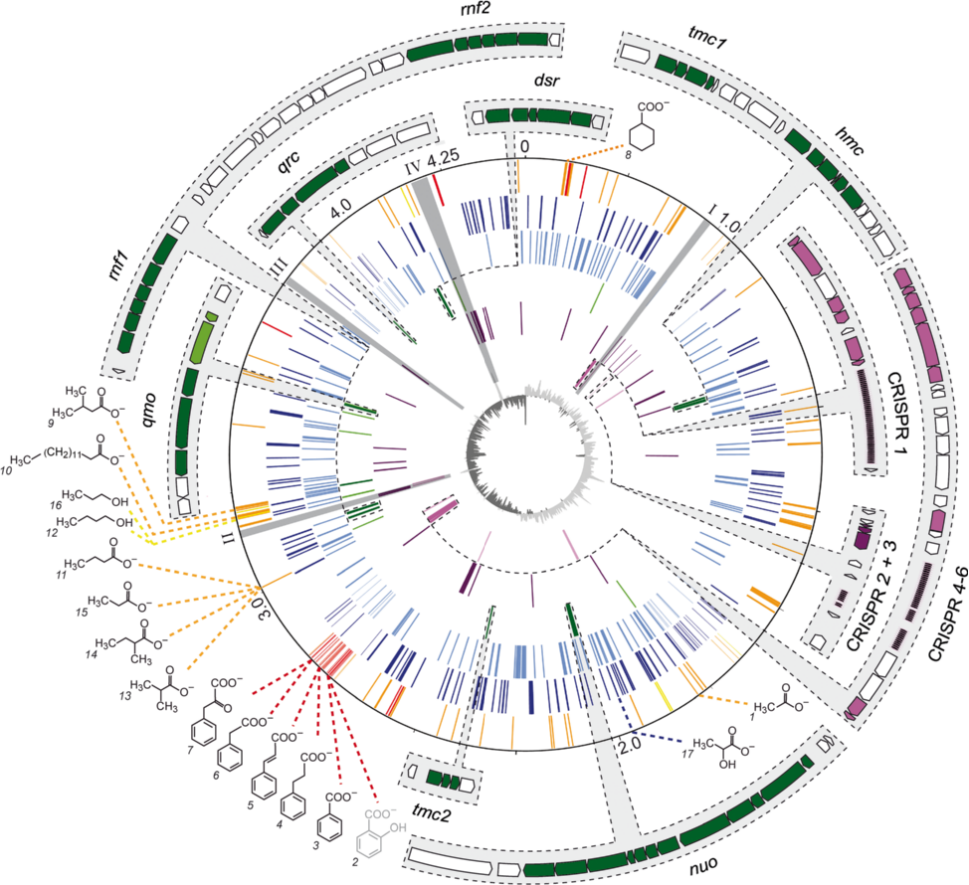
Structural representation of the chromosome of *Desulfococcus multivorans*. Location of gene clusters for aromatic and aliphatic compound degradation pathways are indicated by the corresponding chemical structure, with the respective names described in legend to Fig. 2 (*grey*: genome prediction; *black*: genome prediction confirmed by proteomics). The chromosomal location of redox complexes (*dark green*) and CRISPR-related and CRISPR-associated proteins (*pink*) are enlarged in the peripheral panel. Genomic islands I-IV are indicated by grey shading. Selected gene categories are represented by circles (from outside to inside): (i) aromatic compound degradation (*red*), fatty acid metabolism (*orange*) and alcohol dehydrogenases (*yellow*); (ii) other carbon metabolic functions (*dark blue*); (iii) signal transduction and transcriptional regulation (*light blue*); (iv) membrane-associated redox complexes (*dark green*) and sulfate reduction (*light green*); (v) transposases and mobile elements (*purple*); (vi) CRISPR-related and CRISPR-associated proteins (*pink*) and phage-related (*light pink*); (vii) GC skew (*dark grey*, below average; *light grey*, above average). The scale (Mbp) is indicated by the outer rim

The complete genome of *D. multivorans* consists of a single 4,455,399 bp circular chromosome containing 3,942 ORFs with an average length of 985 bp. The genome size of *D. multivorans* lies in between those of other SRBs such as *Desulfotalea psychrophila* (3.5 Mbp) [19], *Desulfovibrio vulgaris* Hildenborough (3.6 Mbp) [20], and closely related *Desulfococcus oleovorans* strain Hxd3 (3.9 Mbp; GenBank accession: CP000859) on the lower side, and those of *Desulfobacterium autotrophicum* HRM2 (5.6 Mbp) [14] and *Desulfobacula toluolica* Tol2 (5.2 Mbp) [15] on the upper side. General overviews of genomic features of *D. multivorans* are illustrated in Fig. 1 and compared to other genome-sequenced members of the *Desulfobacteraceae* in Table 1.

Three rRNA operons were detected, all of which contain genes for tRNA^Ile^ and tRNA^Ala^ located between the 16S and 23S rRNA genes. Overall, genes of 54 tRNAs for the regular proteinogenic amino acids were identified in the genome. Additionally, genes (*selA*, *selB* and *selD*, respectively) for L-seryl-tRNA selenium transferase, Sec-specific elongation factor and selenophosphate synthase were identified. Five genes harbour the selenocysteine codon UGA upstream of a selenocysteine insertion sequence (SECIS) instructing the ribosome to recognize UGA as Sec codon [21]: heterodisulfide reductase subunit A encoding *dmul_C02770/80*, the δ-subunit of methyl-viologen-reducing hydrogenase (*mvhD*) encoding *dmul_C28840/50*, the α-subunit of formate dehydrogenase (cytoplasmic) encoding *dmul_C16320/30*, selenocysteine-containing peroxiredoxin encoding *dmul_C20060/70*, and BamE encoding *dmul_C24500/10*.

In total, 45 genes related to mobile genetic elements and transposases were discovered in the genome, 11 of which locate in four regions (I, 0.47–0.49 Mbp; II, 3.14–3.16 Mbp; III, 3.78–3.81 Mbp; IV, 4.17–4.21 Mbp) predicted to represent genomic islands (indicated by grey shading in Fig. 1). Most ORFs (56 out of 92) within these islands could not be functionally assigned and were annotated to code for uncharacterized proteins. No prophage was detected, however, the presence of 14 genetic elements related to phage assembly (tail and capsid morphogenesis), phage genome replication and transcription regulation indicates phage impact on the genome of *D. multivorans*.

Six loci containing clustered regularly interspaced palindromic repeats (CRISPR) and 15 genes for CRISPR-associated proteins (Cas) were found in the genome of *D. multivorans* (Fig. 1; purple colored gene clusters). CRISPR loci were recently reported to be present in 40 % of bacterial genomes and > 66 % of the investigated 45 deltaproteobacterial genomes [22]. CRISPR and Cas are considered to constitute an adaptive nucleic acid-based antiviral defense mechanism affiliated to spacer-phage sequence similarity [23, 24] that provides resistance against a particular phage based on a RNA interference mechanism [25]. The CRISPR locus 1 at 0.45 Mbp is closely neighboring a DNA section predicted to represent the genomic island I and thereby resembles the structural relationship observed for other prokaryotic genomes. It contains 83 spacers and genes related to *cas1* and *cas2* (*dmul_C04070/80*) which are required for the integration of new invader-derived spacers [26], *cas6* (*dmul_C04010*) involved in crRNA processing [27, 28], *cas5* and *cas7* (*dmul_C04050/40*) responsible for assembly and surveillance of CRISPR ribonucleoprotein complex (crRNP) as well as *cas3* (*dmul_C04020*) mediating the degradation of DNA targets [29, 30]. The two CRISPR loci (2 and 3) at 0.48 Mbp are considerably shorter and contain 9 and 3 spacers, respectively. No *cas*-related genes were identified in these regions but three genes predicted to code for transposases were found in close proximity. The fourth CRISPR locus at 2.92 Mbp harbours a cluster of *cas1-6* homologs (*dmul_C25810-60*) and a second copy of *cas2* (*dmul_C25940*) upstream to 40 spacers and the fifth CRISPR locus with 10 spacers. The sixth CRISPR locus comprises 18 spacers and homologs of *cas1-2* (*dmul_C26060/70*). The numerous CRISPR systems along with the highly diverse CRISPR spacer content and phage-related elements in the genome is indicative of *D. multivorans*’ exposure to repeated viral impact in its evolutionary history.

### Concept of metabolic reconstruction by proteogenomics

**Fig. 2:**
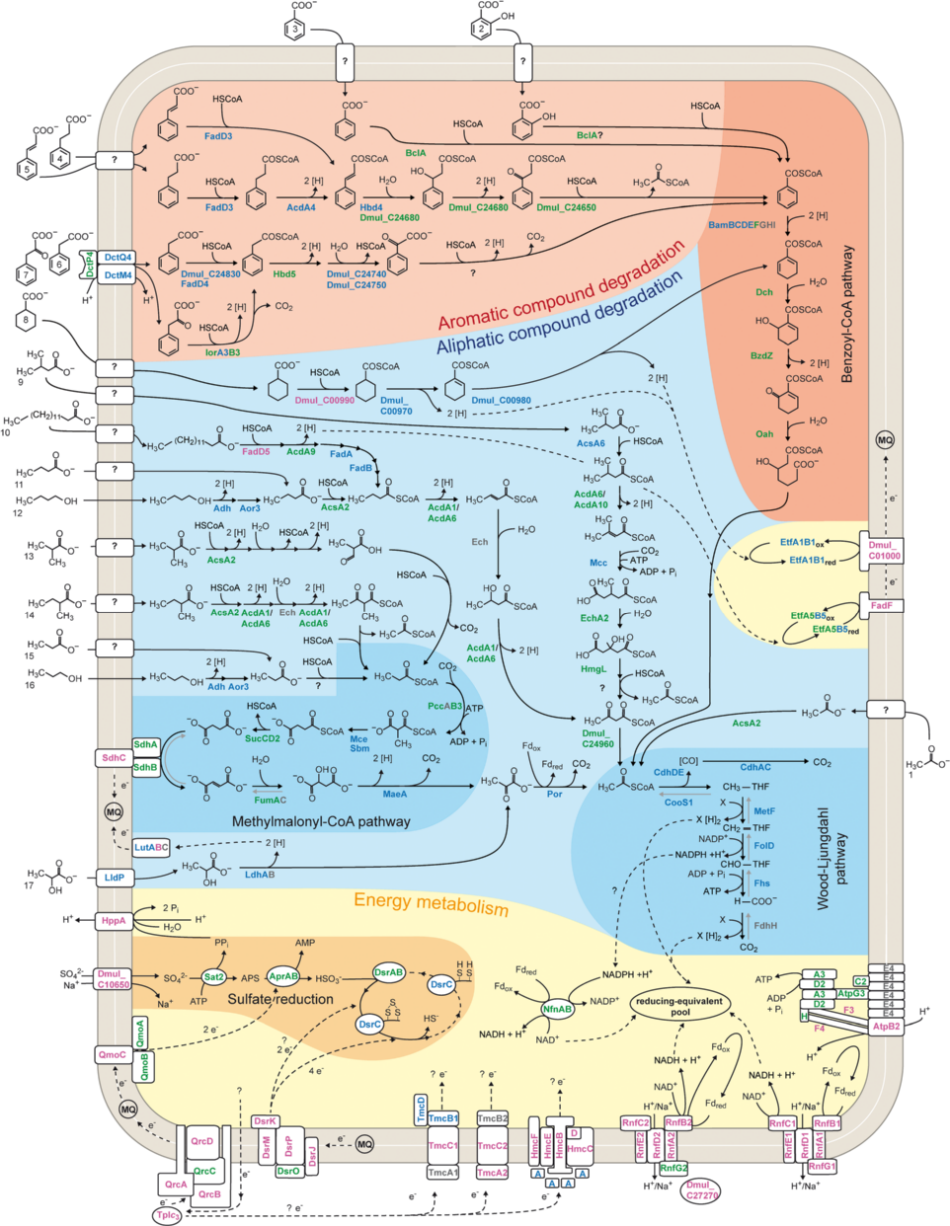
Metabolic reconstruction of *Desulfococcus multivorans* for 17 different growth substrates based on combined genomic and differential proteomic data. Assigned proteins are colour coded as follows: *grey*, genome predicted only; coloured, identified by proteomics (*green*, 2D DIGE; *blue*, shotgun analysis; *pink*, membrane protein-enriched fraction). In case of multiple protein identifications, only one method is indicated hierarchically from 2D DIGE via shotgun analysis down to membrane protein-enriched fraction. Enzyme names and their predicted functions are provided in Additional file 1: Table S1. Putative and assumed routes of electron flow are indicated by dashed lines. Compound names: (1) acetate, (2) 2-hydroxybenzoate, (3) benzoate, (4) 3-phenylpropanoate (hydrocinnamate), (5) cinnamate, (6) phenylacetate, (7) phenylpyruvate, (8) cyclohexane carboxylate, (9) 3-methylbutanoate, (10) myristinate, (11) butanoate, (12) *n*-butanol, (13) isobutanoate, (14) 2-methylbutanoate, (15) propanoate, (16) 1-propanol, and (17) lactate

**Fig. 3:**
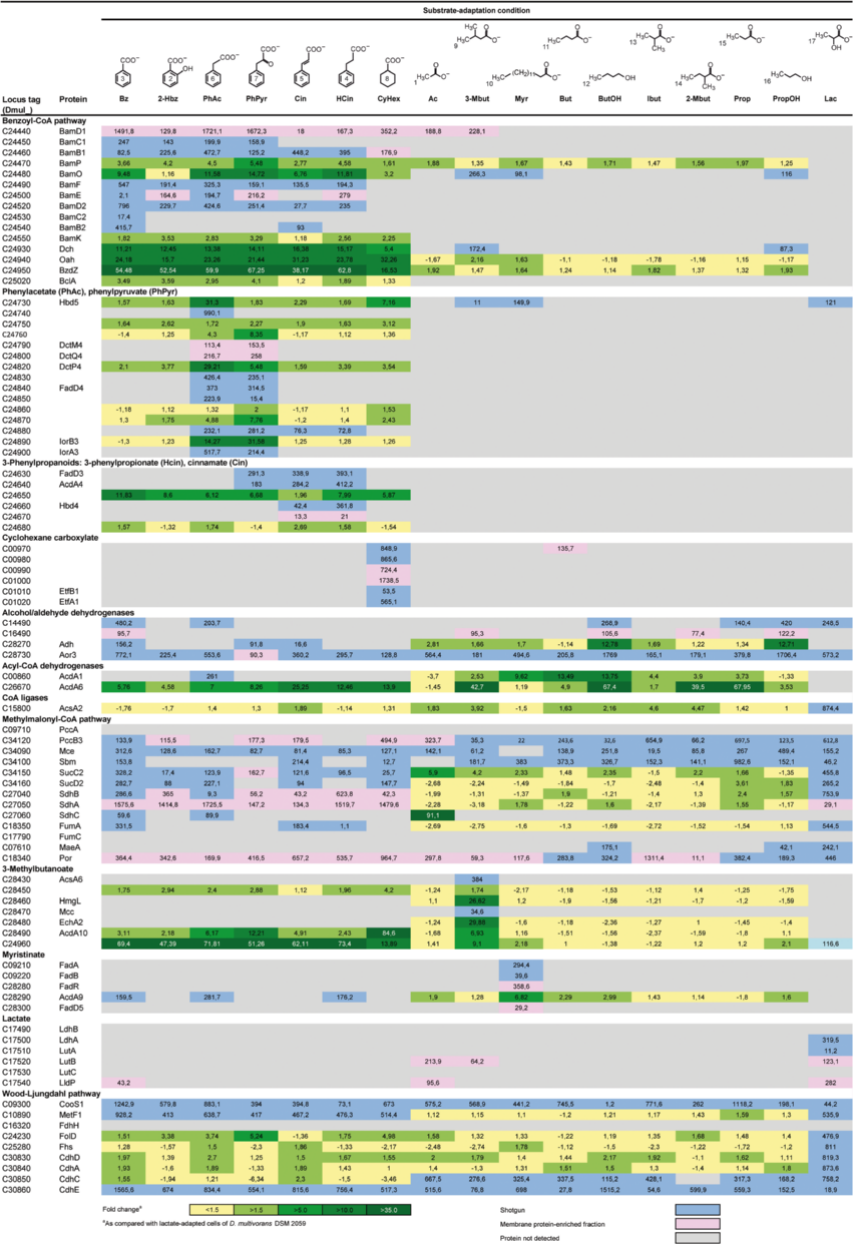
Subproteomes for the degradation of aromatic and aliphatic compounds across different substrate adaptation conditions in *Desulfococcus multivorans*. Compound names are as detailed in legend to Fig. 2. Identified proteins are ordered according to pathways/functional categories and ascending locus tags. Fold changes in protein abundance were determined by 2D DIGE using lactate-adapted cells as reference state. In case of multiple protein identifications, only one method is indicated hierarchically from 2D DIGE (*green*) via shotgun analysis (*blue*) down to membrane protein-enriched fraction (*pink*). Not detected proteins are indicated in grey. Enzyme abbreviations and functional predictions are according to Additional file 1: Table S1

**Fig. 4:**
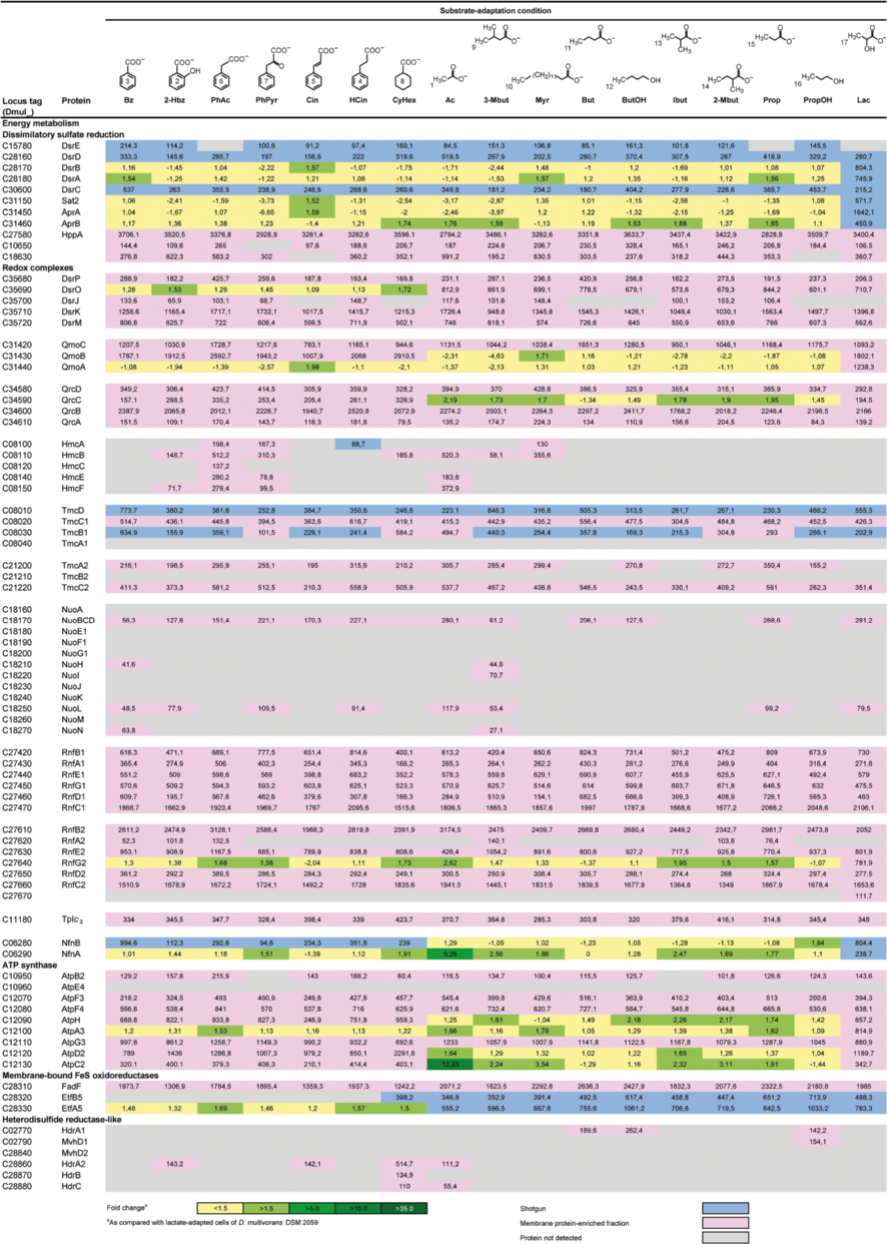
Proteins involved in the energy metabolism of *Desulfococcus multivorans* adapted to aromatic and aliphatic substrate conditions. Compound names are as detailed in legend to Fig. 2. Identified proteins are ordered according to pathways/functional categories and ascending locus tags. Fold changes in protein abundance were determined by 2D DIGE using lactate-adapted cells as reference state in all cases. In case of multiple protein identifications, only one method is indicated hierarchically from 2D DIGE (*green*) via shotgun analysis (*blue*) down to membrane protein-enriched fraction (pink). Not detected proteins are indicated in grey. Enzyme abbreviations and functional predictions are according to Additional file 1: Table S1

**Fig. 5:**
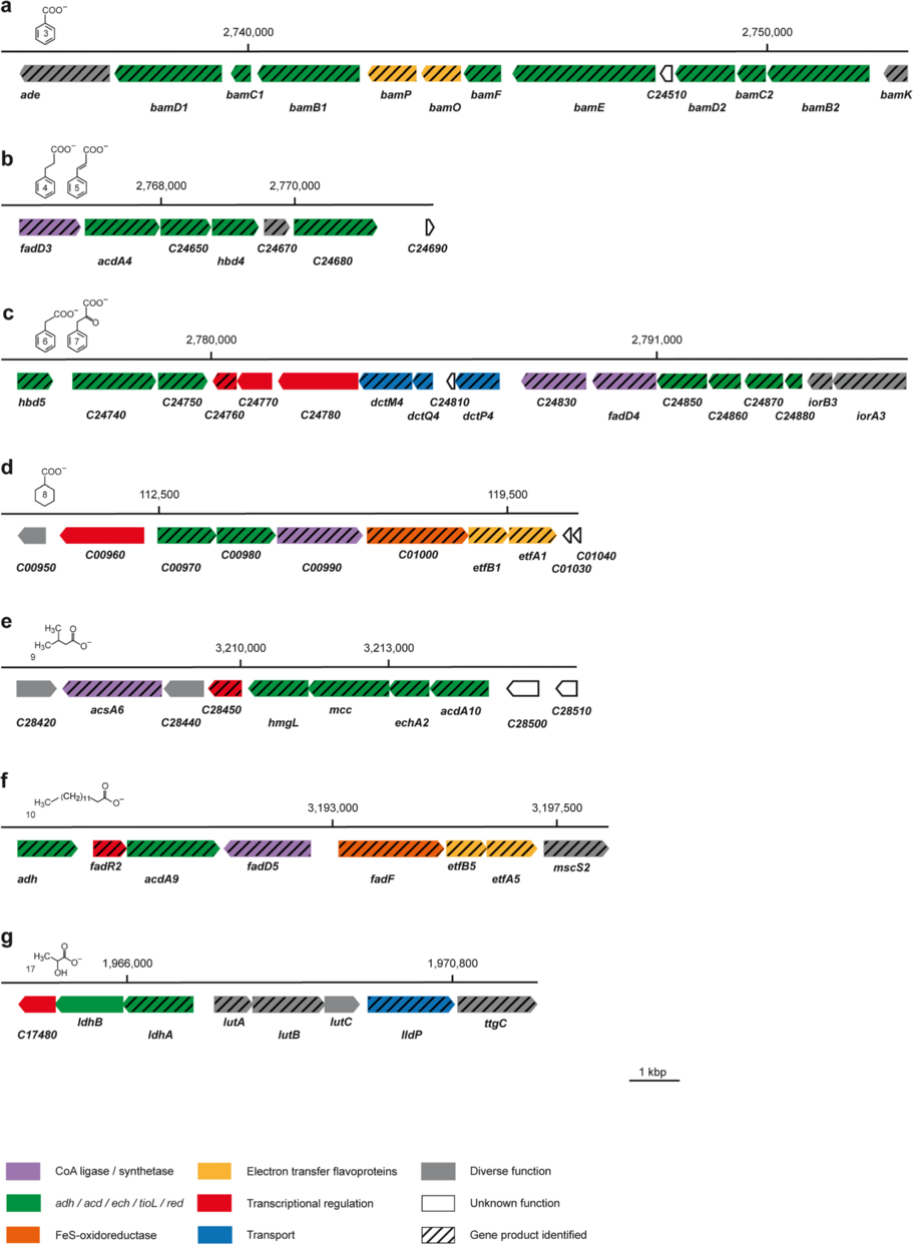
Organization of genes proposed to encode proteins involved in the catabolism of **a** benzoate, **b** 3-phenylpropanoate and cinnamate, **c** phenylacetate and phenylpyruvate, **d** cyclohexane carboxylate, **e** 3-methylbutanoate, **f** myristinate, and **g** lactate. Genes are color coded according their predicted function, as indicated in the insert. Locus tags without gene names are abbreviated *dmul_Cxxxxx*

The present study focused on the metabolic reconstruction of *D. multivorans* since it was originally described as a nutritionally versatile, completely oxidizing SRB [31]. Particular emphasis was on the anaerobic degradation of 6 aromatic (benzoate, 2-hydroxybenzoate, 3-phenylpropanoate, cinnamate, phenylacetate, phenylpyruvate) and 11 aliphatic growth substrates (acetate, 1-propanol, propanoate, *n*-butanol, butanoate, isobutanoate, 2-methylbutanoate, 3-methylbutanoate, lactate, myristinate and cyclohexane carboxylate) ultimately feeding into the Wood-Ljungdahl pathway for terminal oxidation to CO_2_ as well as on the energy metabolism associated with dissimilatory sulfate reduction. Genome-based functional predictions were verified and refined by differential proteome profiling of cells adapted to each of the 17 different substrates. To achieve optimal proteome coverage and consider subcellular localization, 2D DIGE-based profiling of soluble proteins was supplemented by shotgun proteomics and analysis of the membrane protein-enriched fractions. For overview on the proteomic data set and complementarity of the applied proteomics methods refer to Additional file 1: Figure S1. The reconstructed metabolic network is illustrated in Fig. 2, with the underlying proteomic data compiled in Figs. 3 and 4 (see also Additional file 1: Table S1), and selected gene clusters are presented in Fig. 5. The network consists of 170 proteins, 154 of which have been identified (Figs. 3 and 4). Notably, most of the degradation capacities are encoded in a concise genomic region of ~500 kbp surrounding the 3.0 Mbp position, while genes for cytoplasmic and transmembrane electron transfer are dispersed across the genome (Fig. 1). The proteomic coverage of the pathways and protein complexes predicted from the genome and shown in Fig. 2 is as follows: (i) aromatic compound degradation, 27/24 proteins predicted/identified (89 % coverage); (ii) aliphatic compound degradation, 46/39 proteins predicted/identified (85 % coverage); (iii) energy metabolism, 67/67 proteins predicted/identified (100 % coverage).

### Anaerobic degradation of aromatic compounds

Genes assigned to the conversion of 3-phenylpropanoate, cinnamate, phenylacetate and phenylpyruvate to benzoyl-CoA are framed on the upstream side by *bam* genes for reductive dearomatization of benzoyl-CoA and downstream by genes involved in β-oxidation and ring opening, together forming a “catabolic hotspot” on the genome (Fig. 1).

### 3-Phenylpropanoate and cinnamate

Anaerobic degradation of 3-phenylpropanoate (hydrocinnamate) by *D. multivorans* was demonstrated by Widdel [31] and resembles the pathways reported for “*Aromatoleum aromaticum*” EbN1 [32] and *Rhodopseudomonas palustris* [33]. All five enzymes proposed to be involved in 3-phenylpropanoate degradation, are encoded in an operon-like structure (Fig. 5b) and were specifically identified in cinnamate- and 3-phenylpropanoate-adapted cells of *D. multivorans* (Fig. 3). Initial activation of 3-phenylpropanoate (compound 4 in Fig. 2) to 3-phenylpropionyl-CoA (CoA ligase, FadD3) is followed by oxidation to 3-phenyl-2-propenoyl-CoA (acyl-CoA dehydrogenase, AcdA4). Further β-oxidation of the propenoyl-CoA sidechain yielding 3-oxo-3-phenylpropionyl-CoA is then mediated by 3-hydroxybutyryl-CoA dehydrogenase (Hbd4) and 3-hydroxyacyl-CoA dehydrogenase (Dmul_C24680). Finally, thiolytic cleavage forms acetyl-CoA and benzoyl-CoA (thiolase, Dmul_C24650).

Cinnamate (compound 5 in Fig. 2) is most likely directly CoA-activated to 3-phenyl-2-propenoyl-CoA (FadD3) prior to further degradation via the above described pathway for 3-phenylpropanoate. Contrasting the fatty acid CoA-ligase (EbA5317) in “*A. aromaticum*” EbN1, which is also proposed to activate *p*- and *o*-hydroxylated 3-phenylpropanoids to their corresponding CoA-thioesters, FadD3 (22 % sequence identity with EbA5317) may be restricted to non-hydroxylated substrates since growth tests revealed that *D. multivorans* does not utilize *p*-hydroxyphenylpropanoate.

### Phenylacetate and phenylpyruvate

The anaerobic peripheral degradation of phenylacetate (compound 6 in Fig. 2) to benzoyl-CoA was previously described for the denitrifying betaproteobacteria *Thauera aromatica* K172, *Azoarcus evansii* [34, 35] and “*A. aromaticum*” EbN1 [36]. The Pad pathway demonstrated with these three strains involves initial activation to phenylacetyl-CoA (PadJ), subsequent α-oxidation forming phenylglyoxylate (PadBCD) and decarboxylation yielding the central intermediate benzoyl-CoA (PadEFGHI). Genome analysis revealed *D. multivorans* to only possess two genes for PadJ (*dmul_C22990/23060;* 54 and 35 % sequence identity with EbA5402) and two paralogs of *padCD* (*dmul_C32580/70*; 45 and 27 % sequence identity with EbA5395/6, respectively), but no *padEFGHI* genes. The absence of genes for PadB and PadEFGHI indicates these genes not to be required in phenylacetate and phenylpyruvate catabolism of *D. multivorans* which was recently also demonstrated for *D. toluolica* Tol2 [15].

Proteins encoded in 2 adjacent gene clusters at 2.77 Mbp are specifically formed during anaerobic growth with phenylacetate and phenylpyruvate: (i) a TRAP uptake system (DctM4Q4P4) highly similar to the supposedly phenylalanine/phenylacetate importing DctM9Q9P9 system of *D. toluolica* Tol2 (74–90 % identity), (ii) one of two alternative CoA-ligases for phenylacetyl-CoA formation (Dmul_C24830, FadD4), and (iii) a dehydrogenase (Hbd5), a hydratase (Dmul_C24740) as well as a thiolase (Dmul_C24750) for the formation of phenylglyoxylate. Subsequent conversion of phenylglyoxylate to benzoyl-CoA is unclear at present, since the protein abundance profiles do not provide appropriate enzyme candidates.

In case of anaerobic degradation of phenylpyruvate (compound 7 in Fig. 2), substrate-specific formation of a predicted indolepyruvate:ferredoxin oxidoreductase (IorA3B3) may indicate CoA-activation followed by oxidative decarboxylation to phenylacetyl-CoA. Such an activity, converting arylpyruvates to the corresponding arylacetyl-CoA, was described for the archaeon *Pyrococcus furiosus* [37] and postulated for phenylpyruvate degradation in *D. toluolica* Tol2 [15]. In case of the latter, the chromosomal colocalization of *iorA2B2* with *atoAD* and the phenylalanine/phenylacetate-specifically increased abundance in *D. toluolica* Tol2 suggested an involvement of AtoAD in the subsequent reaction from phenylglyoxylate to benzoyl-CoA [15]. However, no *atoAD* genes are present in the genome of *D. multivorans*. Lower level formation of IorA3B3 in phenylacetate-adapted cells may indicate gratuitous induction which was also observed for the orthologs in *D. toluolica* Tol2 [15].

Notably, the second gene cluster contains genes encoding enzymes for the conversion of phenyllactate to phenylpyruvate (Dmul_C24850-70) (Fig. 5c). Indeed, phenyllactate (4 mM) was positively tested (data not shown) to serve as growth substrate for *D. multivorans*. This gene cluster also contains two sensor histidine kinases (Dmul_C24770/80) and a CheY-like transcriptional regulator (Dmul_C24760) of a two-component regulatory system. Therefore, the cluster likely represents a rather complete catabolic module differing from that of denitrifiers and also from the organization as two separate gene clusters in *D. toluolica* Tol2.

### Benzoate and the central benzoyl-CoA pathway

The anaerobic degradation of benzoate (compound 3 in Fig. 2) and most monoaromatic compounds yields the central intermediate benzoyl-CoA [38]. Benzoate is initially activated to benzoyl-CoA by benzoate CoA-ligase (BclA). The BclA protein was identified in *D. multivorans* with all tested aromatic growth substrates (and cyclohexane carboxylate) (Fig. 3), contrasting substrate-specific formation in *D. toluolica* Tol2 [15].

Subsequent anaerobic degradation of benzoyl-CoA proceeds via reductive dearomatization which involves either the ATP-dependent class I benzoyl-CoA reductase (BcrABCD) in facultative anaerobes (e.g., *T. aromatica* K172) [39] or the ATP-independent class II benzoyl-CoA reductase (BamB-I) in obligate anaerobes (e.g., the Fe^III^-reducing deltaproteobacterium *Geobacter metallireducens* GS-15). The BamB subunit is considered to contain the active site of this enzyme complex, forming a heterodimeric structure with BamC, whereas BamDEFGHI are predicted to be involved in ATP-independent electron transfer [40]. As expected, the genome of *D. multivorans* harbours no *bcrABCD* homologues, but one *bam*-cluster at 2.74 Mbp. Interestingly, two copies of each *bamB-D* are present in this cluster separated by *bamOP*, encoding subunits of an electron transfer flavoprotein (Fig. 5a). The two paralogous BamB proteins share 66 % sequence identity with BamB2 being more similar (79 % identity) to the catalytic subunit of *G. metallireducens* GS-15 (BamB1 only 64 %). Accordingly, phylogenetic analysis clusters BamB2 with BamB homologs of *G. metallireducens* GS-15, *D. toluolica* Tol2 (BamB1) and strain NaphS2 (NPH7171) (Additional file 1: Figure S2), suggesting BamB2 to be the active enzyme subunit in ATP-independent reduction of benzoyl-CoA in *D. multivorans*. However, BamB2 was only detected during anaerobic growth with benzoate and cinnamate while BamB1 was detected with all aromatic growth substrates and also cyclohexane carboxylate. Thus, one may speculate that BamB1/2 jointly function in benzoyl-CoA reduction in *D. multivorans*.

Genes for the β-oxidation of the formed cyclohex-1,5-diene-1-carboxyl-CoA, i.e., *dch, bzdZ* and *oah* (*bamQ*), are located downstream of the *bam*-cluster at 2.80 Mbp. Their respective products were detected under all six analyzed aromatic substrate conditions as well as in case of cyclohexane carboxylate and 3-methylbutanoate (Fig. 3).

### Aliphatic compounds

#### Short chain alcohols

The genome of *D. multivorans* harbours five genes (*dmul_C14490*, *16490*, *16540*, *36750* and *adh*) encoding predicted alcohol dehydrogenases. Since the product of only one of them (Adh) was found to be specifically increased in abundance during anaerobic growth with *n*-butanol and 1-propanol (compounds 12 and 16 in Fig. 2), it is likely to be responsible for converting these alcohols to their respective aldehydes. Four aldehyde dehydrogenases (Aor1-3, aldehyde:ferredoxin oxidoreductase; Dmul_14960, NAD-dependent aldehyde dehydrogenase) are encoded in the genome, but only the product of *aor3* was detected under all tested growth conditions (Fig. 3), pointing towards its involvement in aldehyde catabolism in *D. multivorans*.

#### β-Oxidation of unbranched aliphatic carboxylates and cyclohexane carboxylate

*D. multivorans* utilizes fatty acids up to a chain length of C_14_ via classic β-oxidation and cyclohexane carboxylate via the modified β-oxidation route of the central benzoyl-CoA pathway (Fig. 2). Accordingly, the genome contains a wide range of genes associated with β-oxidation-like functions: (i) Seven (3 identified) predicted paralogs of long-chain-fatty-acid-CoA-ligases (FadD) as well as 10 (7 identified) predicted AMP-dependent synthetases and ligases for CoA-activation of carboxylates; (ii) 15 (6 identified) predicted acyl-CoA dehydrogenase (Acd), (iii) 5 (2 identified) predicted enoyl-CoA hydratases (EchA) and (iv) one (identified) predicted 3-hydroxyacyl-CoA dehydrogenase (FadB). Out of these 19 proteins, seven could be assigned to specific pathways as discussed below.

The anaerobic degradation of cyclohexane carboxylate (compound 8 in Fig. 2) was recently elucidated for *G. metallireducens* GS-15. Initial activation to cyclohexanoyl-CoA is followed by 1,2-dehydrogenation to cyclohex-1-ene-1-carboxyl-CoA and subsequent 1,4-dehydrogenation to cyclohex-1,5-diene-1-carboxyl-CoA [41]. Genes (>80 % protein sequence identity to orthologs of *G. metallireducens* GS-15) encoding putative cyclohexanoyl-CoA dehydrogenase (*dmul_C00970*) and cyclohex-1-ene-1-carboxyl-CoA dehydrogenase (*dmul_C00980*) were detected in the genome of *D. multivorans*, directly adjacent to an AMP-dependent synthetase (*dmul_C00990*), a membrane-bound FeS oxidoreductase (*dmul_C01000*) and an electron transfer flavoprotein (*etfA1B1*) (Fig. 5d). All corresponding proteins were specifically detected during anaerobic growth with cyclohexane carboxylate supporting their involvement in this pathway also in case of *D. multivorans*. The formed cyclohex-1,5-diene-1-carboxyl-CoA enters the β-oxidation-like branch of the benzoyl-CoA pathway as supported by specifically increased abundances of respective proteins (Dch, Oah, BzdZ) (Fig. 3).

Myristinate (tetradecanoic acid; compound 10 in Fig. 2) conversion to five acetyl-CoA and one butyryl-CoA is assumed to involve CoA-activation by FadD5, AcdA9-catalyzed dehydrogenation, acetyl-CoA acyltransfer by FadA and oxidation of 3-hydroxyacyl-CoA by FadB, inferred from myristinate-specific protein formation. Respective encoding genes colocalize on a stretch of 10.3 kbp at 3.19 Mbp of the chromosome (Fig. 5f), except for *fadAB* (at 1.03 Mbp). Notably, this genomic locus also includes genes for a transcriptional regulator FadR2 (specifically detected in myristinate-adapted cells) as well as genes *(etfA5B5)* for an electron transfer flavoprotein (ETF) and a constitutively formed membrane-bound FeS oxidoreductase (*fadF*) (Figs. 2 and 3). The latter may be involved in myristinate-specific electron transfer (see below section “Electron transfer flavoproteins”). The TetR-type regulator FadR2 may mediate the observed myristinate-specific protein formation.

Butanoate (compound 11 in Fig. 2) is commonly degraded via crotonyl-CoA to acetoacetyl-CoA which is thiolytically cleaved into two acetyl-CoA. An increased abundance of acetyl-CoA dehydrogenases AcdA1/6 in butanoate- as well as *n*-butanol- and myristinate-adapted cells indicates an involvement of these proteins in conversion of butanoate and unbranched fatty acids in general (Fig. 3). However, none of the many β-oxidation-related genes could be assigned unequivocally to butanoate catabolism based on genomic co-localization and/or substrate-specific product formation, indicating a rather constitutive formation of these enzymes.

Propanoate (compound 15 in Fig. 2) and odd-numbered fatty acids are commonly degraded also via β-oxidation which however yields one propionyl-CoA in addition to the acetyl-CoA moieties. Further degradation of propionyl-CoA proceeds via the methylmalonyl-CoA pathway. Initially, propionyl-CoA is carboxylated to methylmalonyl-CoA, by ATP-dependent propionyl-CoA carboxylase (PccAB3). Methylmalonyl-CoA is transformed from D- to L-conformation by methylmalonyl-CoA epimerase (methylmalonyl-CoA racemase, Mce) prior to being intramolecularly rearranged to succinyl-CoA by methylmalonyl-CoA mutase (Sbm). Succinyl-CoA is further degraded employing enzymes of the tricarboxylic acid cycle (succinyl-CoA ligase, SucCD2; succinate dehydrogenase, SdhABC; fumarate hydratase, FumAC) followed by an oxidative decarboxylation (malic enzyme, MaeA) yielding pyruvate. Genes for all required enzymes are rather scattered across the genome of *D. multivorans* contrasting the clustered organization in *D. autotrophicum* HRM2 [14]. Respective gene products of *D. multivorans* were detected under all aliphatic substrate conditions (except for PccA and FumC) pointing to a constitutive presence of the methylmalonyl-CoA pathway (Figs. 2 and 3).

#### Branched-chain fatty acids

Isobutanoate (compound 13 in Fig. 2) is probably degraded according to the pathway suggested by Stieb and Schink [42] including initial CoA-activation followed by two oxidation and one hydrolysis step(s) yielding methylmalonic semialdehyde. The latter could be subsequently oxidized and decarboxylated forming propionyl-CoA (Fig. 2). Initial CoA-activation of branched-chain fatty acids in *D. multivorans* is probably mediated by AcsA2 (Fig. 3). The unambiguous assignment of genes involved in further conversion was, however, not possible and also no isobutanoate-specific formation of respective proteins was observed.

In case of 2-methylbutanoate (compound 14 in Fig. 2), the methyl-branching is compatible with classical β-oxidation (see short chain alcohols), but thiolytic cleavage of intermediary 2-methylacetoacetyl-CoA yields acetyl-CoA and propionyl-CoA (Fig. 2), with the latter feeding into the methylmalonyl-CoA pathway.

3-Methylbutanoate (isovalerate; compound 9 in Fig. 2) is probably degraded in analogy to the pathway previously proposed for a mixed anaerobic marine co-culture [43]. Initially formed isovaleryl-CoA is oxidized to methylcrotonyl-CoA, which is subsequently carboxylated to methylglutaconyl-CoA, following hydration to 3-hydroxy-3-methylglutaconyl-CoA, and final cleavage into acetoacetyl-CoA and acetyl-CoA. Genome analysis revealed a gene cluster at 3.20 Mbp potentially encoding enzymes for isovalerate degradation as well as a transcriptional regulator (Fig. 5e). Differential proteomics corroborated this prediction by demonstrating exclusive formation of acyl-CoA synthase (AcsA6), enoyl-CoA hydratase (EchA2), methylcrotonyl-CoA carboxylase (Mcc) and 3-hydroxy-3-methylglutaconyl lyase (HmgL) in 3-methylbutanoate-adapted cells of *D. multivorans* (Fig. 3).

#### Lactate and pyruvate

Lactate (compound 17 in Fig. 2) is probably taken up by L-lactate permease (LldP). The latter is encoded in an operon-like structure together with genes for pyruvate-forming lactate dehydrogenase (*ldhAB*), L-lactate utilization proteins (*lutABC*) predicted to transfer electrons from lactate dehydrogenase to membrane-embedded redox partners in *Bacillus subtilis* [44], and a GntR-type transcriptional regulator (*dmul_C17480*) (Fig. 5g). These lactate-related proteins were substrate-specifically formed, supporting their predicted function in *D. multivorans*.

Pyruvate, which is also generated from the C_4_-dicarboxylate malate by malic enzyme (Mae) as the final step of the methylmalonyl-CoA pathway (see section above), is oxidatively decarboxylated to acetyl-CoA by the pyruvate:ferredoxin oxidoreductase (Por). Agreeing with its central metabolic role, Por was found to be constitutively formed.

### Complete oxidation of acetyl-CoA (to CO_2_) via the Wood-Ljungdahl pathway

A catabolic property of *D. multivorans* shared with many members of the *Desulfobacteraceae* is the complete oxidation of acetyl-CoA to CO_2_ via the Wood-Ljungdahl pathway [13]. Genes for this pathway are present in, but dispersed across the genome of *D. multivorans*, contrasting the clustered organization observed for *D. oleovorans* Hxd3, *D. toluolica* Tol2 and *D. autotrophicum* HRM2. Except for formate dehydrogenase (FdhH, solely genome-encoded Fdh) all other protein constituents of the pathway were identified across all tested 17 substrate adaptation conditions, pointing to a constitutive formation. This agrees with proteogenomic data available for *D. autotrophicum* HRM2 [14, 17, 18] and *D. toluolica* Tol2 [15].

Although, homologs of all enzymes of the TCA cycle are encoded in the genome, the cycle is apparently only partially operative within the framework of the methylmalonyl-CoA pathway due to non-detection of aconitase (AcnA), isocitrate dehydrogenase (Icd) and malate dehydrogenase (Mdh) under any of the tested substrate conditions (Figs. 2 and 3).

### Electron transfer flavoproteins

Electron transfer flavoproteins (ETF) are soluble αβ-heterodimeric proteins which serve in accepting electrons from dehydrogenases and transferring them to the membrane (mena)-quinone pool mediated by membrane-bound Etf:quinone oxidoreductases [45]. ETFs can be functionally classified in (i) constitutively formed proteins accepting electrons from a variety of acyl-CoA dehydrogenases and (ii) those being substrate-specifically formed receiving electrons from specific oxidation reactions [46]. EtfAB are predicted in *Syntrophus aciditrophicus* [47], *Syntrophomonas wolfei* [48] and *D. alkenivorans* AK-01 [16] to funnel β-oxidation-derived electrons to the menaquinone-pool. Likewise, electrons liberated during successive dehydrogenation reactions during anaerobic degradation of toluene and *p*-cresol in *D. toluolica* Tol2 are suggested to be conveyed by specific ETF proteins encoded directly adjacent to respective catabolic genes [15].

Seven (five identified) ETF proteins (including BamOP) are encoded in the genome of *D. multivorans*. Three ETF proteins (EtfA2B2, EtfA4B4 and EtfA5B5) were abundantly formed under all analyzed substrate conditions, suggesting a constitutive formation. In case of EtfA5B5 the chromosomally colocalizing membrane-bound FeS-oxidoreductase (FadF) is also present under all tested substrate conditions, suggesting transfer of β-oxidation-generated electrons via EtfA5B5 to the membrane menaquinone pool. The genomes of *D. autotrophicum* HRM2 and *D. oleovorans* Hxd3 also harbour genes for ETFs highly similar (66–80 %) to EtfA5B5 of *D. multivorans*, albeit the respective genomic contexts do not provide any hints on the specific functional roles in these SRB.

The ETF-proteins BamOP were identified under all aromatic substrate conditions, suggesting a specific function in transfer of electrons during reductive dearomatization of benzoyl-CoA. The exclusive detection of EtfA1B1 as well as the associated membrane-bound FeS oxidoreductase Dmul_C01000 in cyclohexane carboxylate-adapted cells indicates their involvement in anaerobic degradation of cyclohexane carboxylate in *D. multivorans* (Figs. 2 and 3).

Overall, the obtained proteogenomic evidence specific for *D. multivorans* as well as *D. toluolica* Tol2 [15] point to an emerging principle of specific ETF-proteins (and associated membrane-bound FeS oxidoreductases) to transfer electrons generated by distinct catabolic cytoplasmic redox reactions to the membrane menaquinone-pool in SRB.

### Dissimilatory sulfate reduction

The energy metabolism of *D. multivorans* centers on dissimilatory sulfate reduction, typical of all SRB. *D. multivorans* is well equipped for the uptake of sulfate, as the genome contains six genes for putative sodium/sulfate symporters and two predicted proton/sulfate symporters (SulP). Products of four of the sodium/sulfate symporters were identified, and two (*Dmul_C10650/18630*) were found to be constitutively formed. The latter are reminiscent of the sulfate uptake systems recently dissected by means of BN-PAGE in *D. toluolica* Tol2 [49] (sequence identities 25–75 %). The product of SulP1 was identified in the membrane protein-enriched fraction of 12 out of the 17 tested substrate conditions, whereas low score detection of SulP2 (Mascot Score 45.8 and 41.4) could indicate the latter to be less relevant for sulfate uptake in *D. multivorans*.

Dissimilatory reduction of sulfate to sulfide proceeds via initial ATP-dependent activation of sulfate to adenylylsulfate (APS; ATP sulfurylase encoded by *sat*), followed by successive reduction of APS to sulfite (APS reductase encoded by *aprAB*) and sulfite to sulfide (dissimilatory sulfite reductase encoded by *dsrAB* and associated proteins *dsrCDE*). The genome of *D. multivorans* contains most of these genes in a region at ~3.50 Mbp: two paralogous *sat* genes at 3.05 and 3.51 Mbp, *aprAB* at 3.54 Mbp, *dsrABD* at 3.17 Mbp, *dsrC* at 3.43 Mbp and *dsrE* at 1.77 Mbp. The non-clustered, but rather scattered distribution of these sulfate reduction genes also occurs in the genomes of *D. vulgaris* Hildenborough, *D. autotrophicum* HRM2 and *D. toluolica* Tol2. Differential proteomics revealed the constitutive and abundant formation of Sat2, AprAB and DsrABCDE of *D. multivorans* under all analyzed substrate conditions (Fig. 4), which was also observed for *D. toluolica* Tol2 under eight different substrate conditions [15], underpinning the essential function of these proteins in SRB energy metabolism.

### Transmembrane redox complexes and cytochromes

#### QmoABC and DsrMKJOP complexes

Among the various described transmembrane complexes in SRB, only the three-subunit, quinone-interacting, membrane-bound oxidoreductase complex QmoABC and the five-subunit DsrMKJOP complex are strictly conserved in currently genome-sequenced SRB [50]. In *D. multivorans* the *qmoABC* and *aprAB* genes colocalize while *dsrMKJOP* and *dsrABC* genes do not. In analogy to other genome sequenced SRB, the QmoABC complex is proposed to channel electrons from the membrane-localized menaquinone pool to AprAB, while the DsrMKJOP performs the analogous function towards DsrAB via soluble DsrC [13, 51]. All QmoABC and DsrMKJOP subunits could be identified in rather high abundance under all tested substrate conditions (Fig. 4), corroborating their predicted essential function in the energy metabolism.

#### HmcABCDEF, TmcABC(D) and QrcABCD complexes

The six-subunit HmcABCDEF [52] complex and the potentially Hmc-derived four-subunit TmcABCD [53] complex often occur in parallel in SRB and are functionally associated with electron transfer between the cytoplasm and the periplasm. While the Tmc complex is assumed to channel electrons from periplasmic hydrogen oxidation to cytoplasmic dissimilatory sulfate reduction, the Hmc complex operates probably in reverse direction to build up reduced conditions in the periplasm [13]. Notably, the genome of *D. multivorans* lacks genes encoding periplasmic hydrogenases (e.g., *hyn*, *hys*, *hyd*), agreeing with the original finding of the strain not being able to utilize hydrogen as an electron donor [31].

The genome of *D. multivorans* encodes a complete Hmc complex at 0.90 Mbp, and formation of several subunits was observed in phenylacetate- (HmcABCEF) as well as phenylpyruvate-adapted (HmcABEF) cells. The comparatively selective and apparently lower-level formation of the Hmc complex indicates an only minor (non-essential) role in general electron transfer, while a potentially specific function in phenylacetate and phenylpyruvate catabolism remains to be demonstrated.

Two paralogous gene clusters for the Tmc complex (*tmcA1B1C1D* at 0.89 Mbp and *tmcA2B2C2* at 2.38 Mbp) are present in the genome of *D. multivorans* sharing 35–64 % (Tmc1) and 27–35 % (Tmc2) amino acid identity with the orthologs from *D. vulgaris* Hildenborough [53]. Hmc complex encoding genes are located downstream of *tmcA1* (Additional file 1: Figure S3) directly adjacent to genes encoding three DNA-binding response regulator proteins (Dmul_C08090/180/210), a DNA-binding universal stress protein (Dmul_C08170) and four signal transduction histidine kinases (Dmul_C08190/200/220/230), resembling the operon-like structure previously described for *D. vulgaris* Hildenborough [53]. In contrast to the above described Hmc complex, most constituents of the two encoded Tmc complexes were identified in the membrane protein-enriched fraction under all 17 substrate adaptation conditions, displaying, however, rather low abundances (Fig. 4). Notably, the periplasmic TmcA1 subunit and cytoplasmic TmcB2 were not detected. While the overall low Tmc complex abundance may render the small (15 kDa) and periplasmic TmcA1 not detectable, the cytoplasmic TmcB2 (50 kDa) is apparently not formed. However, the absence of genes encoding periplasmic formate dehydrogenases and hydrogenases challenges a function of the *D. multivorans* Tmc complexes in the respective electron transfer as described for *D. vulgaris* Hildenborough [53].

The Qrc complex contains three periplasmic (ABC) and one integral membrane (D) subunit(s) and has been associated with connecting periplasmic components, TpI*c*_3_, and non-membrane-anchored hydrogenase or formate dehydrogenase (for overview see [13, 54, 55]). The genome of *D. multivorans* harbours a *qrcABCD* gene cluster at 3.91 Mbp colocalizing with cytochrome biogenesis genes (*ccsAB*); they share high sequence similarities (33–75 % on amino acid level) with their orthologs from *D. vulgaris* Hildenborough [54], *D. autotrophicum* HRM2 [14] and *D. alkenivorans* AK-01 [16]. All four Qrc subunits of *D. multivorans* were abundantly detected in the membrane protein-enriched fraction of all 17 substrate adaptation conditions. The inability of *D. multivorans* to utilize hydrogen and formate, both of which are predicted to serve as periplasmic electron donor for the Qrc complex in *D. vulgaris* Hildenborough [54], suggests a different, though essential function of the Qrc complex in *D. multivorans* energy metabolism. The predicted physiological partner may be the periplasmic TpI*c*_3_ cytochrome (Dmul_C11180).

#### Nuo complex

Genes for the multi-subunit NADH:quinone oxidoreductase complex (Nuo, respiratory complex I; *nuoA-N*) were identified at 2.03 Mbp of the chromosome. Among Deltaproteobacteria, complete *nuo* gene sets seem to be characteristic for *Desulfobulbaceae* and have so far been reported only for seven representatives of *Desulfovibrionaceae* (for overview see [13, 50]). Thus, *D. multivorans* is the first member of *Desulfobacteraceae* possessing the complete *nuo* gene set in a single cluster (Additional file 1: Figure S3). However, only the cytoplasmic fusion protein NuoBCD and the integral membrane subunit NuoL could be detected under most of the tested substrate conditions at rather low abundance (Fig. 4). Hence, the Nuo complex in *D. multivorans* may play only a minor role in energy metabolism with its function still to be resolved.

#### RnfABCDEG complex and Na^+^-based bioenergetics

The Rnf complex is an electron transporting, membrane-localized ferredoxin:NAD^+^-oxidoreductase widespread among Deltaproteobacteria. The six subunit RnfABCDEG complex consists of three integral membrane proteins (RnfADE), two membrane associated (RnfBG) and one soluble subunit(s) (RnfC). It is assumed to link the cellular NADH and ferredoxin pools via bidirectional electron transfer, i.e., (i) oxidation of reduced ferredoxin by RnfB and transfer via RnfDG to RnfC for NAD^+^ reduction coupled to the generation of a Na^+^-gradient, or (ii), vice versa, from NADH to oxidized ferredoxin driven by the membrane potential [50, 56]. Notably, a different type of the Rnf complex was recently reported to be present in *D. toluolica* Tol2 [49]. Here, the RnfB subunit involved in ferredoxin oxidation/reduction is larger (~700 amino acids) as compared to characterized RnfB of *Clostridium ljungdahlii* or *Acetobacterium woodii* (~300 amino acids) and contains NAD^+^-binding sites in addition to the 4Fe-4S cluster and ferredoxin-type iron-sulfur binding domain present in all RnfBs. Furthermore, also the RnfC subunit of *D. toluolica* Tol2 differs, lacking the NADH-ubiquinone oxidoreductase and soluble ligand-binding domains necessitating an RnfC electron acceptor/donor other than NAD^+^/NADH, which has to be identified. Due to these differences, a mode of action different to the classical Rnf complex was suggested [49] that awaits experimental verification.

The genome of *D. multivorans* contains two colocalized *rnf* gene cluster (*rnf1* at 3.09 Mbp and *rnf2* at 3.11 Mbp) encoding an *A. woodii*-type Rnf complex with a small RnfB subunit (Rnf1; 35–55 % amino acid identity) as well as a *D. toluolica* Tol2-type Rnf complex with a large RnfB subunit (Rnf2; 41–84 % amino acid identity), a genetic repertoir similar to *D. autotrophicum* HRM2 [14]. Differential proteomics revealed the formation of all six subunits of Rnf1 and five subunits of Rnf2 under all tested substrate conditions in the membrane protein-enriched fraction; the only exception is the integral membrane protein RnfA2 being only detected under 6 substrate conditions (Fig. 4). The latter may be due to its size and detectability of the resulting tryptic peptides. The cytochrome encoded in the *rnf2* cluster (Dmul_C27670) was only detected in lactate-adapted cells. However, Dmul_C27670 is a small (15 kDa) acidic (pI 5.3) protein, with only few detectable peptides, rendering its identification difficult and implicating its presence may not be completely excluded under the other tested growth substrates.

In contrast to *D. toluolica* Tol2 [15], the genome of *D. multivorans* does not contain genes for a Na^+^-potential-forming NADH:quinone oxidoreductase (NqrA-F).

### Electron bifurcation

Flavin-based electron bifurcation (FBEB) was originally discovered with the butyryl-CoA dehydrogenase complex in *Clostridium kluyveri* and shown to couple the endergonic reduction of ferredoxin with NADH to the exergonic reduction of butyryl-CoA with NADH [57]. Several protein candidates predicted to perform FBEB have been detected also in other anaerobic archaea and bacteria, suggesting this mechanism to be widespread and to play a significant role in cytoplasmic electron transfer of these organisms [50].

#### Hdr/Mvh complex

A protein complex involved in electron bifurcation is the HdrABC/MvhADG complex, which couples the oxidation of H_2_ to the reduction of ferredoxin and heterodisulfide (CoM-S-S-CoB) in methanogens [58]. HdrA contains a FAD-binding site and is therefore predicted to be involved in electron bifurcation/confurcation and to represent the site of ferredoxin reduction. In contrast, the Mvh subunits probably catalyze the H_2_ oxidation (MvhA) and serve in electron transfer (MvhDG) to the FAD centre of HdrA. HdrB catalyzes the reduction of heterodisulfide and is supplied with electrons by HdrC [59]. The *hdrA* gene is highly conserved in methanogenic archaea and orthologs are present in the genomes of a broad range of anaerobic organisms including SRB. Indeed, some SRB genomes harbour a rather large number of *hdrA* orthologs, e.g., *D. autotrophicum* HRM2 (7 orthologs) [14] and *D. toluolica* Tol2 (13 orthologs) [15].

The genome of *D. multivorans* harbours only two *hdrA* homologs (*hdrA1* at 0.32 Mbp and *hdrA2* at 3.25 Mb). The *hdrA1* gene colocalizes with *mvhD1* and pyridine nucleotide-disulphide oxidoreductase (Dmul_C02800), whereas *hdrA2* is located adjacent to *hdrBC* and *mvhD2* similar to the archaean gene organization. Analysis of the membrane protein enriched-fraction of *D. multivorans* revealed rather substrate-specific protein formation: HdrA1 was only detected in cell extracts of cells adapted to anaerobic growth with *n*-butanol, 1-propanol and butanoate, whereas all HdrA2BC subunits were found in cyclohexane carboxylate-adapted cells and HdrA2 was only sporadically observed with other substrate conditions (Fig. 4). These findings agree with the non-detection of HdlA5BC subunits in *D. toluolica* Tol2 and preclude those protein complexes from serving a general function in cytoplasmic electron transfer. They are more likely involved in specifically linking individual redox reactions to the reducing-equivalent pool.

#### NfnAB transhydrogenase

Another protein complex involved in FBEB is the transhydrogenase NfnAB (NADH-dependent reduced ferredoxin:NADP^+^ oxidoreductase). This complex was originally discovered in *Clostridium kluyveri* and reported to catalyze the reduction of ferredoxin with NADPH in the presence of NAD^+^ which was also reduced to NADH [60, 61]. Comparative genome analysis revealed the *nfnAB* genes to be present in many SRB [50]. Deletion of the *nfnAB* genes slightly affected growth of *Desulfovibrio alaskensis* G20 with fumarate and malate, suggesting NfnAB to oxidize NADPH (derived from fumarate and malate conversion) and to reduce NAD^+^ and ferredoxin potentially coupled to energy conservation via Rnf [56].

In *D. multivorans* the *nfnAB* genes are located at 0.69 Mbp of the genome. Both protein subunits were identified in all tested substrate-specific subproteomes, with only NfnA revealing slightly fluctuating abundances (Fig. 4). This constitutive formation indicates a rather central role in the energy metabolism of *D. multivorans* and other Deltaproteobacteria (e.g., *D. alaskensis* G20 [56]). In *D. multivorans*, NfnAB may oxidize NADPH derived from acetyl-CoA oxidation by the Wood-Ljungdahl pathway yielding NADH and reduced ferredoxin (Fig. 2).

### Response to oxygen stress

Besides their frequent detection in anoxic, marine sediments, *Desulfococcus* phylotypes were also reported to be the most abundant among SRB in the photooxic zone of cyanobacterial mats reducing sulfate to sulfide while being exposed to oxygen [62]. Accordingly, the genome of *D. multivorans* contains a plurality of genes coding for oxygen detoxifying enzymes: 4 (1 identified) thioredoxins, 4 (1 identified) peroxidases, 3 (1 identified) superoxide dismutases, 1 (identified) catalase as well as cytochrome oxidase. Additionally, rubredoxin oxidoreductase (Roo1 and Roo2), ruberythrin (Rbr1-5) and desulfoferredoxin (Dfx) may increase the tolerance to oxygen exposure of *D. multivorans.* Detection of at least one protein of each enzyme class (except for cytochrome oxidase) in the subproteomes of all substrate conditions (i.e., constitutive formation) indicates a permanent readiness to cope with sudden oxygen exposure.

### Regulatory potential

The presence of 215 (49 products identified) genes in the genome of *D. multivorans* coding for predicted sensory/regulatory systems indicate the capacity to regulate the metabolic network in a diversified and fine-tuned manner. In comparison, the genomes of *D. autotrophicum* HRM2 and *D. toluolica* Tol2 contain >250 and >430 such genes, respectively [14, 15], providing together first genome-wide evidences for members of the *Desulfobacteraceae* to have the means for comprehensive sensory capacities and regulatory adaptation.

## Conclusions

The genome of *D. multivorans* is rather similar to several other sulfate-reducing Deltaproteobacteria with respect to size, GC content and encoded proteins. The presence of a large number of encoded proteins involved in signal transduction allows *D. multivorans* to adapt to changing environmental conditions as evident from formation of substrate-specific catabolic subproteomes. This flexibility is contrasted by the constitutive and abundant formation of multiple membrane-embedded redox complexes involved in electron transfer to cytoplasmic partners (most importantly sulfate reduction). Notably, specifically formed proteins of electron generating catabolic pathways are connected to the membrane-bound electron transfer system via pathway-specific electron-transfer proteins. The latter are encoded in gene clusters with corresponding catabolic proteins, thus constituting complete degradation modules similar to earlier observations with *D. toluolica* Tol2 [15]. The presence of such modules seems to be a principle to efficiently connect electron generating and consuming processes in completely oxidizing SRB affiliating with *Desulfobacteraceae*, possibly to optimize energy metabolism at the thermodynamic limit.

Abundant and constitutive formation of membrane redox complexes (e.g., QrcABCD), are described in *Desulfovibrio* sp. to connect periplasmic hydrogen and formate oxidation. Despite the genetic and apparent physiological inability of *D. multivorans* to utilize such electron donors, respective membrane redox complexes are abundantly and constitutively formed by this bacterium. This indicates a different, though essential, function of these complexes in its energy metabolism. Furthermore, abundant formation of two most likely functionally different Rnf complexes supposes a prominent role of Na^+^-based bioenergetics for *D. multivorans*. Hence, this bacterium appears to be a catabolic generalist employing a multifunctional and highly interconnected membrane redox complex network.

Furthermore, constitutive formation of a large number of oxygen counter-acting proteins will allow *D. multivorans* to survive oxygen pulses in dynamic marine environments (e.g., tidal systems). The interplay of these characteristics may contribute to its habitat success.

Overall, the study underpins the value of proteomic analysis to enhance functional genomic predictions and represents another puzzle piece in understanding the proteogenomic basis of the habitat-relevance and -success of the deltaproteobacterial SRB family *Desulfobacteraceae*.

## Methods

### Media and cultivation

*Desulfococcus multivorans* DSM 2059 (strain 1be1; originally isolated from a sewage digester (Göttingen, Germany) [31]) was obtained from the Leibniz-Institute DSMZ-German Collection of Microorganisms and Cell Cultures and cultivated under sulfate-reducing conditions in 400 ml flat glass bottles containing defined bicarbonate-buffered brackish water medium [63]. Cultures were incubated at 28 °C with the following growth substrates (in alphabetic order; concentration in mM is given in parenthesis): acetate (30), benzoate (3), *n*-butanol (5), butanoate (5), cinnamate (3), cyclohexane carboxylate (3), 2-hydroxybenzoate (2), isobutanoate (5), lactate (10), 2-methylbutanoate (5), 3-methylbutanoate (5), myristinate (3), phenylacetate (4), 3-phenylpropanoate (3), phenylpyruvate (3), 1-propanol (7) and propanoate (15). Adaptation of *D. multivorans* over at least five passages to the individual substrate conditions and cell harvest were performed as described recently [15].

### DNA sequencing, assembly and annotation

DNA was isolated with the Genomic DNA kit (Qiagen, Hildesheim, Germany) according to the manufacturer’s instructions. Recombinant plasmid and fosmid shotgun libraries were constructed. Plasmid libraries with average insert sizes of 1.5 and 2.5 kbp were generated from sonified DNA [64]. Additionally, a fosmid library was constructed (84-fold physical coverage) for data finishing and assembly confirmation (Epicentre Technologies, Madison, WI, USA). Templates for sequencing were obtained by insert amplification via PCR or by plasmid isolation. Sequencing was carried out using ABI3730XL capillary systems (ABI). In total, 107,083 sequencing reads were generated and resulted in 15-fold sequencing coverage. Sequence quality assessment and assembly were performed with a quality of less than 1 error in 100,000 bases using PHRAP (Phragment assembly program 1999 (http://www.phrap.org/phredphrapconsed.html)) and Consed [65]. Structural rRNAs and tRNAs were determined using RNAmmer [66] and tRNAscan-SE [67]. Protein-coding sequences (CDS) were predicted by the ORF-finding program Glimmer3 [68] and manually revised and curated using Artemis (v.12.0) [69]. The generated ORF dataset was screened against nonredundant protein databases (SWISSPROT, TREMBL) and the genome was manually annotated applying the annotation platform HTGA (High Throughput Gene Annotation; [70]). The genome sequence of *Desulfococcus multivorans* DSM 2059 has been submitted to GenBank under the BioProject PRJNA310394 with accession number CP015381.

### Profiling of soluble proteins by 2D DIGE and protein identification by MALDI-TOF-MS/MS

Extracts of soluble proteins of *D. multivorans* were prepared and 2D DIGE performed essentially as reported previously [71]. Cell pellets (approx. 100 mg wet weight) from 4 biological replicates per substrate condition were suspended in lysis buffer (7 M urea, 2 M thiourea, 30 mM Tris/HCl, 4 % CHAPS, pH 8.5), cell breakage was achieved with the PlusOne sample grinding kit (GE Healthcare, Munich, Germany) and the protein concentration was determined according to the method of Bradford [72]. For minimal labelling, 200 picomoles of CyDye DIGE fluors (GE Healthcare) were used to label 50 μg of protein sample. Protein extracts of lactate-adapted cells served as reference state and were labelled with Cy5. Protein extracts from the other 16 substrate adaptation conditions represented the test states and were each labelled with Cy3. The internal standard contained equal amounts of all test and the reference state(s) and was labelled with Cy2. Per gel, 50 μg each of the labelled reference state, test state and internal standard were applied. To account for biological variation [73], four parallel gels were run with labelled protein extracts from four individual cultures (i.e., biological replicates) for each test state. First dimension separation by isoelectric focusing (IEF) was conducted with 24 cm-long IPG strips (pH 3–11 NL; GE Healthcare) run in an IPGphor system (GE Healthcare). The IEF program used was as follows: 30 V for 7 h, 60 V for 6 h, 200 V for 1 h, 1,000 V for 1 h, gradual gradient to 8,000 V within 0.5 h and 8,000 V for 60,000 Vhs. Second dimension separation of proteins according to molecular size was done by SDS-PAGE (12.5 % gels, v/v) using an EttanDalt*twelve* system (GE Healthcare).

2D DIGE gels were scanned directly after completion of electrophoresis with a Typhoon 9400 scanner (GE Healthcare). Cropped gel images were analyzed with the DeCyder software (version 7.0; GE Healthcare) in two different work packages: one for aromatic (incl. cyclohexane carboxylate) and the other for aliphatic substrates. Parameters for spot detection were as described previously [36]. Three of the four biological replicates were included for the reference and each test state. Changes in the protein abundance of ≥ **|**1.5**|**-fold were regarded as significant [73]. Separate preparative colloidal Coomassie Brilliant Blue (cCBB)-stained gels were run (300 μg protein load) to obtain sufficient amounts of protein for reliable mass spectrometric identification [36]. Spots of interest were excised using the EXQuest spot cutter (Bio-Rad) from two cCBB-stained gels per analysed substrate state, and subsequently washed and tryptically digested as described recently [74].

Sample digests were spotted onto Anchorchip steel targets (Bruker Daltonik GmbH, Bremen, Germany) and analysed with an UltrafleXtreme MALDI-TOF/TOF mass spectrometer (Bruker Daltonik GmbH) as recently described [74]. Peptide mass fingerprint (PMF) searches were performed with a Mascot server (version 2.3) against the translated genome of *D. multivorans* with a mass tolerance of 25 ppm. Five lift spectra were collected to confirm PMF identification and three additional spectra were acquired of unassigned peaks applying feedback by the ProteinScape platform (version 3.1; Bruker Daltonik GmbH). In case of failed PMF identification, eight lift spectra of suitable precursors were acquired. MS/MS searches were performed with a mass tolerance of 100 ppm. For both, MS and MS/MS searches, Mascot scores not meeting the 95 % certainty criterion were not considered significant. A single miscleavage was allowed (enzyme trypsin) and carbamidomethyl (C) and oxidation (M) were set as fixed and variable modifications, respectively.

### Analysis of membrane protein-enriched fraction

Total membrane protein fractions were prepared from 2 biological replicates per substrate condition and analyzed as recently reported [74]. Essentially, cell extracts generated by French Press® (Sim-Aminco Ltd, Rochester, NY, USA) were treated with ice-cold carbonate and hot SDS to solubilize the membrane proteins. Protein content was determined with the RC-DC kit (Bio-Rad), and protein separation was achieved using 12.5 % SDS mini gels (10 × 7 cm; Bio-Rad). Each sample lane (10 μg protein load) was divided into 4 gel slices, and each slice cut into smaller pieces (about 1 mm^3^) prior to washing, reduction, alkylation and tryptic digest [74]. Separation and mass spectrometric analysis of peptides was performed with a nano-LC system (UltiMate 3000; Dionex GmbH, Germering, Germany) equipped with a 25 cm analytical column (C18, 2 μm bead size, 75 μm inner diameter; ThermoFisher Scientific) operated in a trap-column mode (C18, 5 μm bead size, 2 cm length, 75 μm inner diameter) using a 120 min linear gradient [74]. The nano-LC eluent was continuously analyzed by an online-coupled electrospray (captive spray; Bruker Daltonik GmbH) ion-trap mass spectrometer (amaZon speed ETD; Bruker Daltonik GmbH). The instrument was operated in positive mode with a capillary current of 1.3 kV and drygas flow of 3 l/min at 150 °C. Active precursor exclusion was set for 0.2 min. Per full scan MS, 20 MS/MS spectra of the most intense masses were acquired. Protein identification was performed with ProteinScape as described above, allowing for a mass difference of 0.4 Da and applying a target decoy strategy (false discovery rate < 1 %).

### Shotgun proteomic analysis

For shotgun analysis, cell pellets from 3 biological replicates per substrate condition were suspended in lysis buffer (7 M urea, 2 M thiourea, 30 mM Tris/HCl, pH 8.5). Cell breakage, removal of cell debris, reduction with dithiotreitol, alkylation with iodoacetamide, and tryptic in-solution digest were performed as previously described [74]. Separation of total peptide mixtures per sample was performed by nano-LC-ESI-MS/MS (see above section) applying a linear 240 min gradient. Protein identification was performed via the ProteinScape platform (see above section).

## Additional files

Additional file 1: Figure S1. Distribution of the 1,307 detected proteins by 2D DIGE, whole cell shotgun analysis and preparation of the membrane protein-enriched fraction of *D. multivorans* grown with 17 different substrates. Figure S2 Phylogenetic relationship of the class II benzoyl-CoA reductase catalytic subunit BamB and other uncharacterized aldehyde: ferredoxin oxidoreductases (AFOR) of selected Deltaproteobacteria. Figure S3 Scale model and chromosomal localization of transmembrane redox complex containing genes of *D. multivorans*. Table S1 Listing of locus tags of genes manually assigned to metabolic pathways and energy conservation as displayed in Figs. 2 and 3. (PDF 433 kb)

## Abbreviations

FBEB: Flavin-based electron bifurcation
SRB: Sulfate-reducing bacteria
