# Supplementary material for: Genome and catabolic subproteomes of the marine, nutritionally versatile, sulfate-reducing bacterium Desulfococcus multivorans DSM 2059
Source: BMC Genomics. 2016 Nov 15;17:918. doi: 10.1186/s12864-016-3236-7 (PMC5109826; doi:10.1186/s12864-016-3236-7)
Supplement: Additional file 1: Figure S1. — Distribution of the 1,307 detected proteins by 2D DIGE, whole cell shotgun analysis and preparation of the membrane protein-enriched fraction of D. multivorans grown with 17 different substrates. Figure S2 Phylogenetic relationship of the class II benzoyl-CoA reductase catalytic subunit BamB and other uncharacterized aldehyde: ferredoxin oxidoreductases (AFOR) of selected Deltaproteobacteria. Figure S3 Scale model and chromosomal localization of transmembrane redox complex containing genes of D. multivorans. Table S1 Listing of locus tags of genes manually assigned to metabolic pathways and energy conservation as displayed in Figs. 2 and 3. (PDF 433 kb) [file 12864_2016_3236_MOESM1_ESM.pdf]

Supporting information
for

Genome and catabolic subproteomes of the marine, nutritionally versatile, sulfate-reducing bacterium *Desulfococcus multivorans* DSM 2059

Marvin Dörries, Lars Wöhlbrand, Michael Kube, Richard Reinhardt, Ralf Rabus

Content

Figure S1:
Proteomic data set of *D. multivorans* 2

Figure S2:
Phylogenetic relationship of the class II benzoyl-CoA reductase catalytic subunit BamB and other uncharacterized aldehyde:ferredoxin oxidoreductases (AFOR) of selected δ-proteobacteria 3

Figure S3:
Scale model and chromosomal localization of transmembrane redox complex containing genes of *D. multivorans* 4

Table S1:
Listings of locus tags of genes assigned to metabolic pathways and energy conservation of *D. multivorans* 5

For correspondence: E-mail: rabus@icbm.de; Tel. (+49) 441 798-3884; Fax (+49) 441 798-3404

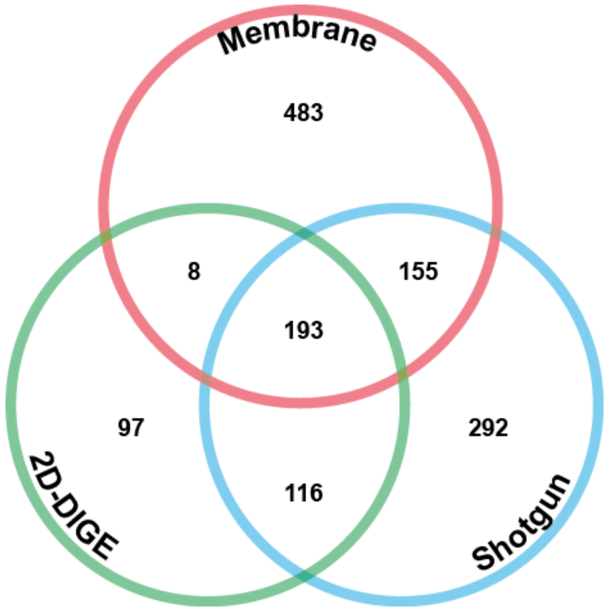

Figure S1: Proteomic data set of *D. multivorans*. In the present study, a total of 1307 different proteins could be identified applying a combination of quantitative 2D DIGE analysis together with whole cell shotgun analysis and preparation of the membrane protein-enriched fraction. Proteome analysis by 2D DIGE was performed in two stages comprised of (i) aromatic (incl. cyclohexane carboxylate) or (ii) aliphatic substrates – cells adapted to growth with lactate served as reference in both cases. Out of 340 identified proteins, 277 significantly changed abundance (56 only with aromatic, 124 only with aliphatic substrates and 97 in all substrate conditions). 193 proteins were identified under all conditions with all applied methods. Overall, 97 proteins (out of 340) were only detected by 2D DIGE, 292 (out of 756) only with the shotgun approach and 483 (out of 839) only in the membrane protein-enriched samples, demonstrating complementarity of the applied proteomic methods.

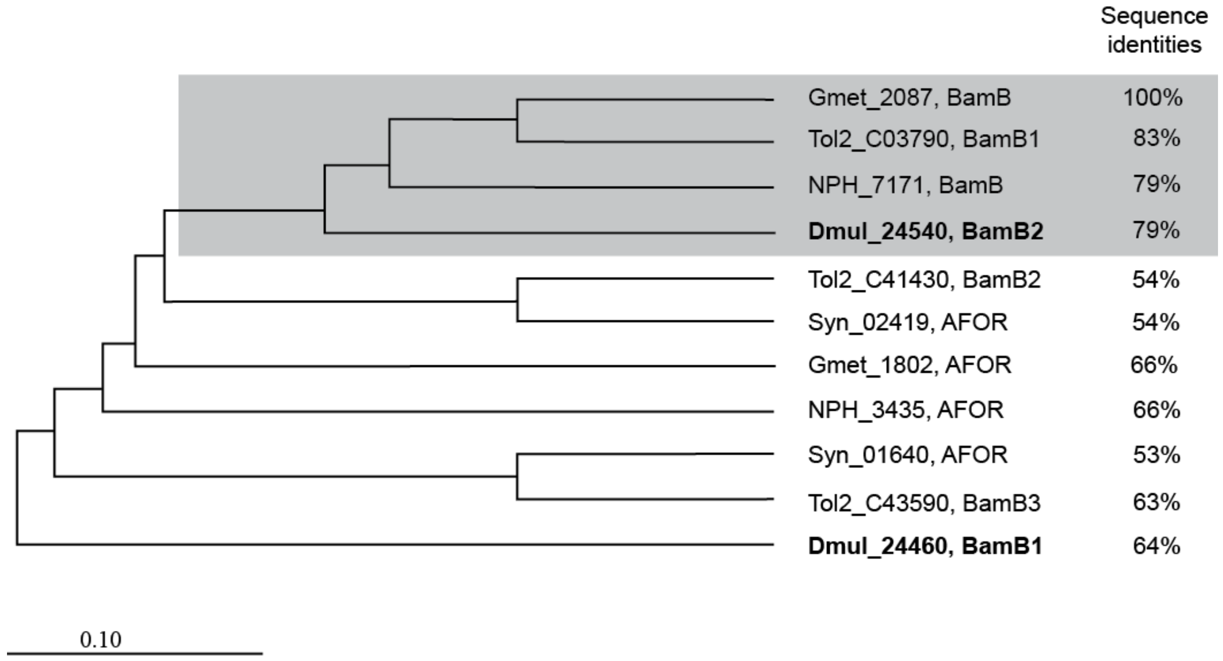

Figure S2: Phylogenetic relationship of the class II benzoyl-CoA reductase catalytic subunit BamB and other uncharacterized aldehyde:ferredoxin oxidoreductases (AFOR) of selected delta-proteobacteria; Dmul, *D. multivorans*; Tol2, *D. toluolica* Tol2; Gmet, *G. metallireducens* GS-15; NPH, strain NaphS2; Syn, *S. aciditrophicus* SB. Protein sequences were aligned applying the ClustalW method, calculations were conducted with the RaxML (version 7.03) and the „rapid bootstrap analysis“ algorithm (500 iterations). The scale bar indicates that 0.1 of the branch length represents 10% estimated amino acid sequence divergence. Sequence identities refer to pBLAST searches against the ORF-set of *D. multivorans* (compared to Gmet_2087, BamB).

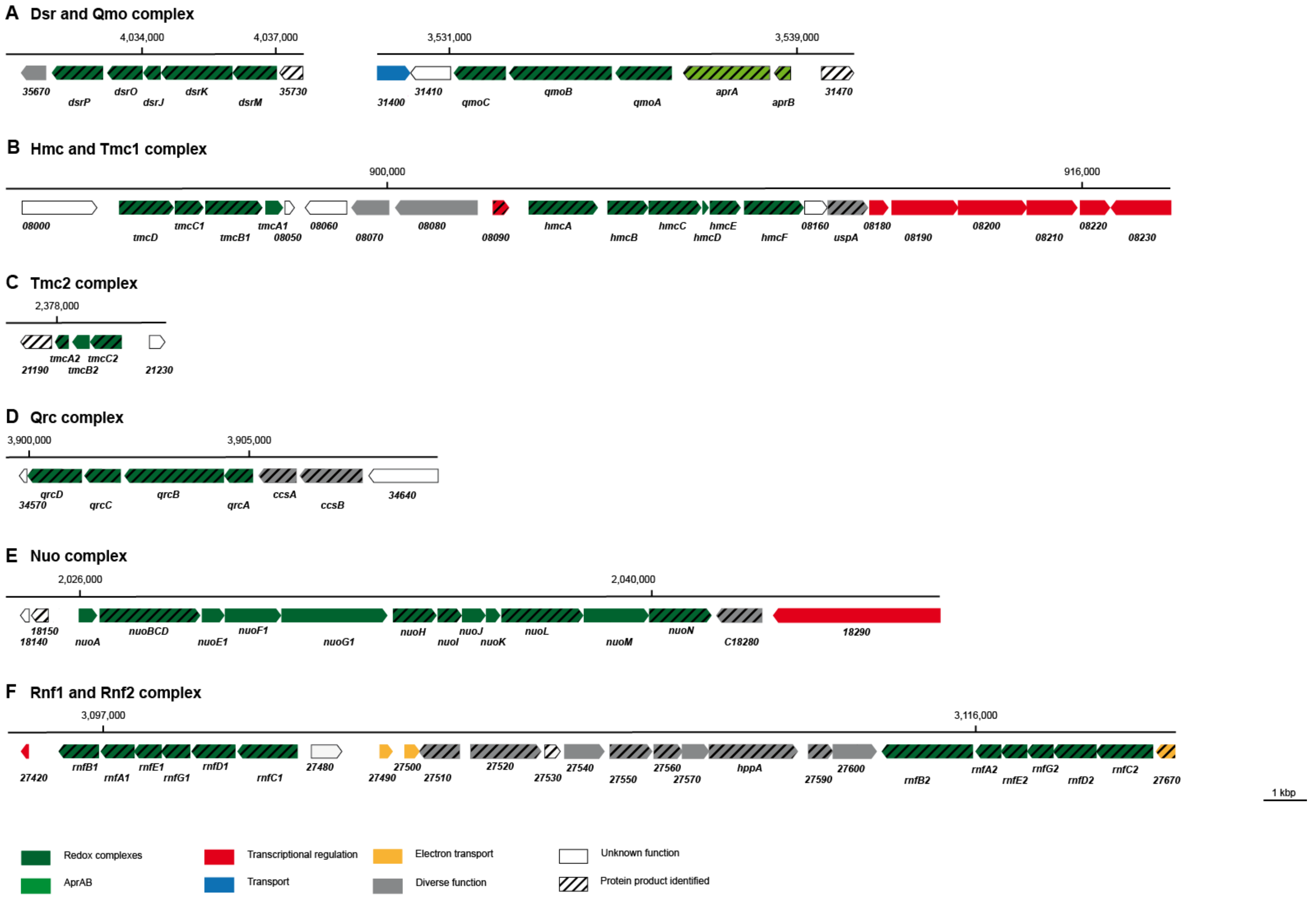

Figure S3: Scale model and chromosomal localization of transmembrane redox complex containing genes of *D. multivorans*. Distinct gene functions are colour-coded. Locus tags without gene names are abbreviated *dmul_XXXXX*.

Table S1: Listings of locus tags of genes assigned to metabolic pathways and energy conservation of *D. multivorans*

Locus_tag	Gene_name	Protein	Length (aa)	Start (bp)	Stop (bp)	Strand (F/R)
**Benzoyl-CoA pathway**						
Dmul_24440	*bamD1*	BamD1: uncharacterized iron-sulfur cluster binding protein	702	2739614	2737509	R
Dmul_24450	*bamC1*	BamC1: 4Fe-4S ferredoxin iron-sulfur domain protein	123	2740153	2739785	R
Dmul_24460	*bamB1*	BamB1: tungsten-containing aldehyde:ferredoxin oxidoreductase	665	2742269	2740275	R
Dmul_24470	*bamP*	BamP: electron transfer flavoprotein, alpha subunit	319	2743366	2742410	R
Dmul_24480	*bamO*	BamO: electron transfer flavoprotein, beta subunit	260	2744215	2743436	R
Dmul_24490	*bamF*	BamF: coenzyme F420 non-reducing hydrogenase, delta subunit	238	2744969	2744256	R
Dmul_24500	*bamE*	BamE: predicted heterodisulfide reductase, iron-sulfur subunit A	930	2747976	2745187	R
Dmul_24520	*bamD2*	BamD2: putative heterodisulfide reductase, subunit B	383	2749454	2748306	R
Dmul_24530	*bamC2*	BamC2: 4Fe-4S ferredoxin	181	2750035	2749493	R
Dmul_24540	*bamB2*	BamB2: aldehyde:ferredoxin oxidoreductase	667	2752057	2750057	R
Dmul_24550	*bamK*	BamK: predicted GCN5-related N-acetyltransferase, associated with benzoate catabolic cluster	150	2752779	2752330	R
Dmul_24930	*dch*	Dch: enoyl-CoA hydratase/isomerase related to dienoyl-CoA hydratase	255	2801985	2802749	F
Dmul_24940	*oah*	Oah: predicted 6-oxo-cyclohex-1-ene-carbonyl- CoA hydrolase	377	2803036	2804166	F
Dmul_24950	*bzdZ*	BzdZ: predicted dehydrogenase	249	2804182	2804928	F
Dmul_25020	*bclA*	BclA: benzoate-CoA ligase	516	2810644	2812191	F
**Phenylacetate (PhAc), Phenylpyruvate (PhPyr)**						
Dmul_24730	*hbd5*	Hbd5: 3-hydroxybutyryl-CoA dehydrogenase	284	2775175	2776026	F
Dmul_24740		fusion protein (3-hydroxyacyl-CoA dehydrogenase and enoyl-CoA hydratase/isomerase family protein)	680	2776548	2778587	F
Dmul_24750		thiolase	396	2778684	2779871	F
Dmul_24760		two component system transcriptional regulator	185	2780595	2780041	R
Dmul_24790	*dctM4*	DctM4: C4-TRAP dicarboxylate transport system permease	430	2784952	2783663	R
Dmul_24800	*dctQ4*	DctQ4: C4-TRAP dicarboxylate transporter	158	2785448	2784975	R
Dmul_24820	*dctP4*	DctP4: C4-TRAP dicarboxylate transporter periplasmic binding protein	359	2787120	2786044	R

Dmul_24830		AMP-dependent synthetase and ligase	526	2789283	2787706	R
Dmul_24840	*fadD4*	FadD4: long-chain-fatty-acid--CoA ligase	518	2791039	2789486	R
Dmul_24850		predicted R-phenyllactate dehydratase subunit alpha	416	2792306	2791059	R
Dmul_24860		predicted R-phenyllactate dehydratase activator	269	2793121	2792315	R
Dmul_24870		putative phenylpyruvate reductase	319	2794189	2793233	R
Dmul_24880		ACT domain protein	144	2794654	2794223	R
Dmul_24890	*iorB3*	IorB3: indolepyruvate oxidoreductase subunit beta	196	2795398	2794811	R
Dmul_24900	*iorA3*	IorA3: indolepyruvate oxidoreductase subunit alpha	602	2797243	2795438	R
**3-Phenylpropanoids: 3-phenylpropionate (Hcin), cinnamate (Cin)**						
Dmul_24630	*fadD3*	FadD3: long-chain-fatty-acid--CoA ligase	499	2763295	2764791	F
Dmul_24640	*acdA4*	AcdA4: acyl-CoA dehydrogenase	607	2764938	2766758	F
Dmul_24650		thiolase	396	2766821	2768008	F
Dmul_24660	*hbd4*	Hbd4: 3-hydroxybutyryl-CoA dehydrogenase	374	2768076	2769197	F
Dmul_24670		conserved uncharacterized protein	201	2769365	2769967	F
Dmul_24680		fusion protein (3-hydroxyacyl-CoA dehydrogenase and enoyl-CoA hydratase/isomerase family protein)	680	2770111	2772150	F
**Cyclohexane carboxylate**						
Dmul_00970		cyclohex-1-carbonyl-CoA dehydrogenase	380	112492	113631	F
Dmul_00980		cyclohex-1-ene-1-carbonyl-CoA dehydrogenase	380	113672	114811	F
Dmul_00990		AMP-dependent synthetase and ligase	556	114881	116548	F
Dmul_01000		iron-sulfur cluster-binding oxidoreductase	663	116666	118654	F
Dmul_01010	*etfB1*	EtfB1: electron transfer flavoprotein, subunit beta	257	118687	119457	F
Dmul_01020	*etfA1*	EtfA1: electron transfer flavoprotein, subunit alpha	306	119493	120410	F
**Alcohol/Aldehyde dehydrogenases**						
Dmul_14490		iron-containing alcohol dehydrogenase	372	1646306	1645191	R
Dmul_16490		alcohol dehydrogenase, GroES domain protein	318	1857346	1858299	F
Dmul_28270	*adh*	Adh: iron-containing alcohol dehydrogenase	388	3187198	3188361	F
Dmul_14960		NAD-dependent aldehyde dehydrogenase	513	1700505	1702043	F
**Acyl-CoA dehydrogenases**						
Dmul_00860	*acdA1*	AcdA1: acyl-CoA dehydrogenase	604	100621	98810	R

Dmul_26670	*acdA6*	AcdA6: acyl-CoA dehydrogenase	385	3011036	3012190	F
Dmul_28490	*acdA10*	AcdA10: acyl-CoA dehydrogenase	382	3214918	3213773	R
**CoA ligases**						
Dmul_15800	*acsA2*	AcsA2: acetyl-coenzyme A synthetase (acetate--CoA ligase)	587	1785690	1787450	F
**Methylmalonyl-CoA pathway**						
Dmul_09170	*pccA*	PccA: propionyl-CoA carboxylase, subunit alpha	669	1087392	1085386	R
Dmul_34120	*pccB3*	PccB3: propionyl-CoA carboxylase, subunit beta	517	3844645	3846195	F
Dmul_34090	*mce*	Mce: methylmalonyl-CoA racemase	134	3840708	3841109	F
Dmul_34100	*sbm*	Sbm: methylmalonyl-CoA mutase-like	713	3841143	3843281	F
Dmul_34150	*sucC2*	SucC2: succinyl-CoA ligase, subunit gamma	388	3848870	3850033	F
Dmul_34160	*sucD2*	SucD2: succinyl-CoA ligase, subunit alpha	289	3850072	3850938	F
Dmul_27040	*sdhB*	SdhB: succinate dehydrogenase and fumarate reductase iron-sulfur protein	255	3057091	3056327	R
Dmul_27050	*sdhA*	SdhA: succinate dehydrogenase flavoprotein subunit	637	3059001	3057091	R
Dmul_27060	*sdhC*	SdhC: succinate dehydrogenase/fumarate reductase cytochrome b subunit	213	3059656	3059018	R
Dmul_18350	*fumA*	FumA: fumarate hydratase class I (fumarase)	535	2058597	2056993	R
Dmul_17790	*fumC*	FumC: fumarate hydratase class II (fumarase C)	466	1992822	1994219	F
Dmul_07610	*maeA*	MaeA: NAD-dependent malic enzyme	552	840749	842404	F
Dmul_18340	*por*	Por: pyruvate:ferredoxin oxidoreductase	1215	2056611	2052967	R
**3-Methylbutyrate**						
Dmul_28430	*acsA6*	AcsA6: acetyl-coenzyme A synthetase (acetate--CoA ligase)	650	3208412	3206463	R
Dmul_28450		transcriptional regulator, HTH-type	212	3210007	3209372	R
Dmul_28460	*hmgL*	HmgL: hydroxymethylglutaryl-CoA lyase	390	3211339	3210170	R
Dmul_28470	*mcc*	Mcc: methylcrotonyl CoA carboxylase	530	3212948	3211359	R
Dmul_28480	*echA2*	EchA2: enoyl-CoA hydratase/isomerase	260	3213732	3212953	R
Dmul_24960		Acetyl-CoA acetyltransferase	388	2804994	2806157	F
**Myristinate**						
Dmul_09210	*fadA*	FadA: 3-ketoacyl-CoA thiolase (Beta-ketothiolase) (Acetyl-CoA acyltransferase)	394	1030768	1029587	R
Dmul_09220	*fadB*	FadB: 3-hydroxyacyl-CoA dehydrogenase, NAD-binding	801	1033203	1030801	R

Dmul_28280	*fadR2*	FadR2: transcriptional regulator, TetR family	213	3188704	3189342	F
Dmul_28290	*acdA9*	AcdA9: acyl-CoA dehydrogenase	604	3189380	3191191	F
Dmul_28300	*fadD5*	FadD5: long-chain-fatty-acid--CoA ligase	565	3193006	3191312	R
**Lactate**						
Dmul_17490	*ldhB*	LdhB: lactate dehydrogenase, subunit beta	443	1965982	1964654	R
Dmul_17500	*ldhA*	LdhA: lactate dehydrogenase, subunit alpha	461	1967370	1965988	R
Dmul_17510	*lutA*	LutA: L-lactate utilization protein A	249	1967802	1968548	F
Dmul_17520	*lutB*	LutB: L-lactate utilization protein B	473	1968544	1969962	F
Dmul_17530	*lutC*	LutC: L-lactate utilization protein C	224	1969958	1970629	F
Dmul_17540	*lldP*	LldP: L-lactate permease	569	1970814	1972520	F
**Wood-Ljungdahl pathway**						
Dmul_09300	*cooS1*	CooS1: carbon monoxide dehydrogenase , catalytic subunit	658	1041186	1039213	R
Dmul_10890	*metF1*	MetF1: methylenetetrahydrofolate reductase	307	1220264	1219344	R
Dmul_16320	*fdhA*	FdhA: formate dehydrogenase, subunit alpha	528	1839994	1838411	R
Dmul_24230	*folD*	FolD: bifunctional protein folD [includes: methylenetetrahydrofolate dehydrogenase + methenyltetrahydrofolate cyclohydrolase	303	2715544	2714636	R
Dmul_25280	*fhs*	Fhs: formate--tetrahydrofolate ligase	587	2846186	2847946	F
Dmul_30830	*cdhD*	CdhD: CO dehydrogenase/acetyl-CoA synthase complex, delta subunit	529	3457813	3459399	F
Dmul_30840	*cdhA*	CdhA: CO dehydrogenase, catalytic subunit	672	3459471	3461486	F
Dmul_30850	*cdhC*	CdhC: CO dehydrogenase/acetyl-CoA synthase complex, subunit beta	737	3461563	3463773	F
Dmul_30860	*cdhE*	CdhE: CO dehydrogenase/acetyl-CoA synthase complex, subunit gamma	448	3463891	3465234	F
**Energy metabolism**						
**Dissimilatory sulfate reduction**						
Dmul_15780	*dsrE*	DsrE family protein	116	1784397	1784050	R
Dmul_28160	*dsrD*	DsrD: sulfite reductase, dissimilatory-type, subunit delta	80	3178262	3178023	R
Dmul_28170	*dsrB*	DsrB: sulfite reductase, dissimilatory-type, subunit beta	382	3179480	3178335	R
Dmul_28180	*dsrA*	DsrA: sulfite reductase, dissimilatory-type, subunit alpha	74	3180516	3180737	F
Dmul_30600	*dsrC*	DsrC: sulfite reductase, dissimilatory-type, gamma subunit	105	3431330	3431016	R
Dmul_31150	*sat2*	Sat2: sulfate adenylyltransferase (ATP sulfurylase)	423	3505633	3504365	R

Dmul_31450	*aprA*	AprA: adenylylsulfate reductase, subunit alpha	657	3538355	3536385	R
Dmul_31460	*aprB*	AprB: adenylylsulfate reductase, subunit beta	124	3538825	3538454	R
Dmul_27580	*hppA*	HppA: membrane-bound proton-translocating pyrophosphatase (H(+)-PPase)	669	3109904	3111910	F
Dmul_10650		sodium/sulfate symporter	495	1194071	1192587	R
**Redox complexes**						
Dmul_35680	*dsrP*	DsrP: predicted redox complex linked to DsrAB, transmembrane subunit	385	4033119	4031965	R
Dmul_35690	*dsrO*	DsrO: predicted redox complex linked to DsrAB, iron-sulfur binding subunit	265	4034025	4033231	R
Dmul_35700	*dsrJ*	DsrJ: predicted redox complex linked to DsrAB, cytochrome c subunit	129	4034414	4034028	R
Dmul_35710	*dsrK*	DsrK: predicted redox complex linked to DsrAB , iron-sulfur binding subunit	540	4036050	4034431	R
Dmul_35720	*dsrM*	DsrM: predicted redox complex linked to DsrAB, transmembrane subunit	333	4037055	4036057	R
Dmul_31420	*qmoC*	QmoC: quinone interacting membrane bound oxidoreductase, subunit C	382	3532323	3531178	R
Dmul_31430	*qmoB*	QmoB: quinone interacting membrane bound oxidoreductase, subunit B	778	3534746	3532413	R
Dmul_31440	*qmoA*	QmoA: predicted quinone interacting membrane bound oxidoreductase, subunit A	425	3536120	3534846	R
Dmul_34580	*qrcD*	QrcD: quinone reductase complex, subunit delta	410	3901262	3900033	R
Dmul_34590	*qrcC*	QrcC: quinone reductase complex, subunit gamma	272	3902149	3901334	R
Dmul_34600	*qrcB*	QrcB: quinone reductase complex, subunit beta	753	3904507	3902249	R
Dmul_34610	*qrcA*	QrcA: predicted quinone reductase complex, subunit alpha	215	3905178	3904534	R
Dmul_08100	*hmcA*	HmcA: high-molecular-weight cytochrome c (cytochrome CC3)	543	903312	904940	F
Dmul_08110	*hmcB*	HmcB: HMC redox complex, transmembrane protein	318	905163	906116	F
Dmul_08120	*hmcC*	HmcC: HMC redox complex, integral membrane protein	409	906119	907345	F
Dmul_08140	*hmcE*	HmcE: HMC redox complex, integral membrane protein	229	907534	908220	F
Dmul_08150	*hmcF*	HmcF: HMC redox complex, cytoplasmic iron-sulfur protein	464	908302	909693	F
Dmul_08010	*tmcD*	TmcD: predicted tmc redox complex, uncharacterized protein	425	893840	895114	F

Dmul_08020	*tmcC1*	TmcC1: predicted tmc redox complex, uncharacterized membrane protein	219	895132	895788	F
Dmul_08030	*tmcB1*	TmcB1: tmc redox complex, cytoplasmic iron-sulfur protein	445	895834	897168	F
Dmul_08040	*tmcA1*	TmcA1: predicted tmc redox complex, acidic cyctchrome c, class III family protein	132	897235	897630	F
Dmul_21200	*tmcA2*	TmcA2: predicted Tmc redox complex, acidic cytochrome c, class III family protein	137	2378224	2377814	R
Dmul_21210	*tmcB2*	TmcB2: predicted Tmc redox complex, cytoplasmic iron-sulfur protein	127	2378644	2378264	R
Dmul_21220	*tmcC2*	TmcC2: predicted Tmc redox complex, uncharacterized membrane protein	236	2379376	2378669	R
Dmul_18160	*nuoA*	NuoA: NADH quinone oxidoreductase, subunit A	134	2026992	2027393	F
Dmul_18170	*nuoBCD*	NuoBCD: NADH quinone oxidoreductase, subunits B C D	764	2027468	2029759	F
Dmul_18180	*nuoE2*	NuoE2: NADH quinone oxidoreductase, subunit E	169	2029811	2030317	F
Dmul_18190	*nuoF2*	NuoF2: NADH-quinone oxidoreductase, subunit F	423	2030326	2031594	F
Dmul_18200	*nuoG2*	NuoG2: NADH quinone oxidoreductase, subunit G	801	2031604	2034006	F
Dmul_18210	*nuoH*	NuoH: NADH quinone oxidoreductase, subunit H	328	2034142	2035125	F
Dmul_18220	*nuoI*	NuoI: NADH quinone oxidoreductase, subunit I	178	2035167	2035700	F
Dmul_18230	*nuoJ*	NuoJ: NADH quinone oxidoreductase, subunit J	175	2035713	2036237	F
Dmul_18240	*nuoK*	NuoK: NADH quinone oxidoreductase, subunit K	104	2036237	2036548	F
Dmul_18250	*nuoL*	NuoL: NADH quinone oxidoreductase, subunit L	626	2036588	2038465	F
Dmul_18260	*nuoM*	NuoM: NADH quinone oxidoreductase, subunit M	492	2038481	2039956	F
Dmul_18270	*nuoN*	NuoN: NADH quinone oxidoreductase, subunit N	471	2039959	2041371	F
Dmul_27420	*rnfB1*	RnfB1: electron transport complex protein RnfB	303	3096928	3096020	R
Dmul_27430	*rnfA1*	RnfA1: electron transport complex protein RnfA	254	3097744	3096983	R
Dmul_27440	*rnfE1*	RnfE1: predicted electron transport complex protein RnfE	207	3098371	3097751	R
Dmul_27450	*rnfG1*	RnfG1: electron transport complex protein RnfG	218	3099031	3098378	R
Dmul_27460	*rnfD1*	RnfD1: electron transport complex protien RnfD	330	3100062	3099073	R
Dmul_27470	*rnfC1*	RnfC1: predicted electron transport complex protein RnfC	454	3101486	3100125	R

Dmul_27610	*rnfB2*	RnfB2: electron transport complex protein RnfB	691	3115926	3113854	R
Dmul_27620	*rnfA2*	RnfA2: electron transport complex protein RnfA	191	3116574	3116002	R
Dmul_27630	*rnfE2*	RnfE2: electron transport complex protein RnfE	197	3117178	3116588	R
Dmul_27640	*rnfG2*	RnfG2: electron transport complex protein RnfG	197	3117787	3117197	R
Dmul_27650	*rnfD2*	RnfD2: electron transport complex protien RnfD	327	3118773	3117793	R
Dmul_27660	*rnfC2*	RnfC2: electron transport complex protein RnfC	431	3120061	3118769	R
Dmul_27670		cytochrome c, class III family protein	145	3120569	3120135	R
Dmul_11180	*tpIc*$_3$	predicted cyctchrome c, class III family protein	233	1250229	1250927	F
Dmul_06280	*nfnA*	NfnA: NADH-dependent reduced ferredoxin:NADP+ oxidoreductase, subunit alpha	455	692313	690949	R
Dmul_06290	*nfnB*	NfnB: NADH-dependent reduced ferredoxin:NADP+ oxidoreductase, subunit beta	311	693303	692371	R
**ATP synthase**						
Dmul_10950	*atpB2*	AtpB2: ATP synthase, subunit alpha (ATP synthase F0 sector subunit alpha)	229	1223936	1224622	F
Dmul_10960	*atpE4*	AtpE4: ATP synthase subunit gamma (ATP synthase F0 sector subunit gamma)	93	1224735	1225013	F
Dmul_12070	*atpF3*	AtpF3: ATP synthase, subunit beta (ATP synthase F0 sector subunit beta)	141	1343901	1344323	F
Dmul_12080	*atpF4*	AtpF4: ATP synthase, subunit beta (ATP synthase F0 sector subunit beta)	208	1344383	1345006	F
Dmul_12090	*atpH*	AtpH: ATP synthase, subunit delta (ATP synthase F1 sector subunit delta)	183	1345009	1345557	F
Dmul_12100	*atpA3*	AtpA3: ATP synthase subunit alpha	505	1345560	1347074	F
Dmul_12110	*atpG2*	AtpG2: ATP synthase, subunit gamma (ATP synthase F1 sector subunit gamma)	297	1347093	1347983	F
Dmul_12120	*atpD2*	AtpD2: ATP synthase, subunit beta (ATP synthase F1 sector subunit beta)	471	1348028	1349440	F
Dmul_12130	*atpC2*	AtpC2: ATP synthase, subunit epsilon (ATP synthase F1 sector subunit epsilon)	138	1349471	1349884	F
**Membrane-bound FeS oxidoreductases**						
Dmul_28310	*fadF*	FadF: uncharacterized protein fadF	690	3193595	3195664	F

Dmul_28320	*etfB5*	EtfB5: electron transfer flavoprotein, subunit beta	261	3195739	3196521	F
Dmul_28330	*etfA5*	EtfA5: electron transfer flavoprotein, subunit alpha	324	3196521	3197492	F
**Heterodisulfide reductase-like**						
Dmul_02770	*hdrA1*	HdrA1: heterodisulfide reductase-like protein, iron-sulfur subunit	610	321829	320000	R
Dmul_02790	*mvhD1*	MvhD1: methyl-viologen-reducing hydrogenase, subunit delta	528	324084	322501	R
Dmul_28840	*mvhD2*	MvhD2: methyl-viologen-reducing hydrogenase, delta subunit	58	3248351	3248178	R
Dmul_28860	*hdrA2*	HdrA2: CoB--CoM heterodisulfide reductase, iron-sulfur subunit alpha	1007	3251779	3248759	R
Dmul_28870	*hdrB*	HdrB: CoB--CoM heterodisulfide reductase, iron-sulfur subunit beta	293	3252650	3251772	R
Dmul_28880	*hdrC*	HdrC: CoB--CoM heterodisulfide reductase, iron-sulfur subunit gamma	210	3253299	3252670	R
